# Drivers of within-host genetic diversity in acute infections of viruses

**DOI:** 10.1371/journal.ppat.1009029

**Published:** 2020-11-04

**Authors:** Maoz Gelbart, Sheri Harari, Ya’ara Ben-Ari, Talia Kustin, Dana Wolf, Michal Mandelboim, Orna Mor, Pleuni S. Pennings, Adi Stern

**Affiliations:** 1 The Shmunis School of Biomedicine and Cancer Research, George S. Wise Faculty of Life Sciences, Tel Aviv University, Tel Aviv, Israel; 2 Clinical Virology Unit, Hadassah Hebrew University Medical Center, Jerusalem, Israel; 3 The Lautenberg Center for General and Tumor Immunology, IMRIC, the Faculty of Medicine, the Hebrew University, Jerusalem, Israel; 4 Central Virology Laboratory, Ministry of Health, Sheba Medical Center, Ramat-Gan, Israel; 5 Department of Epidemiology and Preventive Medicine, School of Public Health, Sackler Faculty of Medicine, Tel Aviv University, Tel Aviv, Israel; 6 Sackler School of Medicine, Tel Aviv University, Tel Aviv, Israel; 7 Department of Biology, San Francisco State University, San Francisco, California, United States of America; Emory University School of Medicine, UNITED STATES

## Abstract

Genetic diversity is the fuel of evolution and facilitates adaptation to novel environments. However, our understanding of what drives differences in the genetic diversity during the early stages of viral infection is somewhat limited. Here, we use ultra-deep sequencing to interrogate 43 clinical samples taken from early infections of the human-infecting viruses HIV, RSV and CMV. Hundreds to thousands of virus templates were sequenced per sample, allowing us to reveal dramatic differences in within-host genetic diversity among virus populations. We found that increased diversity was mostly driven by presence of multiple divergent genotypes in HIV and CMV samples, which we suggest reflect multiple transmitted/founder viruses. Conversely, we detected an abundance of low frequency hyper-edited genomes in RSV samples, presumably reflecting defective virus genomes (DVGs). We suggest that RSV is characterized by higher levels of cellular co-infection, which allow for complementation and hence elevated levels of DVGs.

## Author summary

The few days or weeks following infection with a virus, termed acute infection, are critical for virus establishment. Here we sought to characterize what leads to differences in the genetic diversity of different viruses sampled during acute infection. We performed ultra-deep sequencing of hundreds to thousands viral genomes from forty-three samples spanning three pathogenic human viruses: HIV, RSV and CMV. We found major differences in the genetic diversity of these different viruses, and in different patients infected with the same virus. We investigated the factors responsible for these differences. We found that the DNA virus CMV was less diverse, most likely since it has a lower mutation rate than the RNA viruses HIV and RSV. We also found that the samples with the highest genetic diversity, which included one CMV sample and two HIV samples, bore evidence for multiple genotype infection. In other words, patients from whom these samples were taken were infected with two different “strains” of the virus. Finally, we also found evidence that viral genomes of HIV, and in particular RSV, are edited by the innate immune system of the host, leading to the presence of defective virus genomes.

## Introduction

Viruses are among the fastest evolving entities on earth. Thanks to short generation times, large population sizes and high mutation rates, viruses and in particular RNA viruses rapidly accumulate genetic diversity. This genetic diversity is key to successful adaptation of viruses to novel challenges such as the immune system and drugs [1]. The short time window following virus transmission determines whether viruses are able to establish a successful infection, and when this occurs, the initial infection is termed *acute infection*. These first few days to weeks of viral replication may go unnoticed, since they sometimes precede symptoms. On the genetic side, the within-host genetic diversity of viruses during acute infections is naturally much lower than the within-host genetic diversity of viruses during persistent infection [2]. This is due to two reasons: first, the longer the infection, the more time there is for accumulation of neutral genetic diversity, and second, the adaptive immune response, which often leads to an increase in viral genetic diversity in the form of immune escape variants, doesn’t occur until a few weeks post infection. Much remains unknown regarding the genetic diversity of viruses during acute infection: how many different genotypes found an infection? What is the role of standing genetic diversity in escape from the immune system, or evasion of drugs? And how does cell-autonomous innate immunity affect the genetic diversity of a viral population? To answer these questions, deep population sequencing is necessary, i.e., accurate sequencing that maintains high yield and allows sequencing a large number of viral genomes, and allows the study of haplotypes rather than isolated mutational events.

While many innovative accurate sequencing approaches have been developed recently, most are inapplicable for sequencing of clinical samples where the initial biomass is very low, e.g. [3,4,5]. One notable exception, called Primal-Seq, is based on a multiplexed amplicon approach [6]. While extremely powerful for many uses, it requires the design of multiple primers (often up to hundreds), which can be problematic when the strain of the virus is unknown, or when regions of the genome are divergent. We developed an approach that is tailored for deep population sequencing of samples from acute infection of different viruses, and includes a bioinformatics approach for the inference of divergent viral haplotypes from the sequencing data. We validate and characterize the pros and cons of our sequencing approach. We sequence 43 samples from three different major human pathogenic viruses: human immunodeficiency virus (HIV), respiratory syncytial virus (RSV), and cytomegalovirus (CMV), all sampled during the acute infection stage. We compare the within-host genetic diversity among and within different virus populations, and find patterns characteristic of each virus. We demonstrate the role of multiple transmitted/founder viruses as major contributors to the genetic diversity in HIV and CMV during the early acute stage of an infection. Furthermore, we identify and quantify the impact of various host editing enzymes on the mutational spectrum of viral genomes *in vivo*. Intriguingly, we find that RSV samples bear high levels of potentially defective virus genome (DVGs) as compared to the two other viruses analyzed herein.

## Results

### Probing an accurate sequencing approach

We sought to develop a combined molecular biology and bioinformatics approach for sequencing clinical samples from diverse viruses. To this end, we combined several concepts that have been used previously, including high-yield and high-fidelity polymerases, sequencing error reduction through overlapping paired end reads, and minimization of template loss across different stages of the protocol [6–10]. We further developed a method for inferring haplotypes based on enrichment of mutations shared on the same read (Materials and Methods). We investigated in depth the performance of our approach, dubbed AccuNGS, on synthetically created mixes of DNA and RNA. While AccuNGS sequencing errors accrued on average at a rate of 10^−5^ for DNA and 10^−4^ for RNA (S1 Fig, S1 and S2 Tables), we also found that this average error rate may be misleading: the smaller the number of genomes sequenced, the larger the variance in errors (S2 Fig), in line with previous works [6,11–13]. This leads us to caution against inferences made on individual mutation frequencies, especially those lower than ~1%, as noted previously [6]. However, we could conclude that AccuNGS is useful for inferring aggregated measures of diversity, and found that AccuNGS inferences of diversity were substantially lower when compared to a more standard sequencing approach (S1D Fig). We also tested the performance of our haplotype inference tool on the synthetic data, and found that it can detect the presence of divergent low frequency haplotypes, contingent on high enough coverage (S3 Fig, S1 Text).

### In depth sequencing of different virus populations during acute/early infections

We next set out to sequence viruses from clinical samples. We initially obtained a total of 46 samples from patients recently infected by the RNA viruses HIV and RSV, and the DNA virus CMV (Table 1, S3 Table). An important consideration when performing sequencing is to estimate how many templates were actually sequenced. One way to do this is to add a barcode (also called a primer-ID, or a unique molecular identifier UMI) during library preparation, which can later on allow counting barcodes to estimate the number of sequenced genomes [14] (S1 Text). We evaluated the barcoding approach on our synthetic RNA samples, and found that we sequenced on average 10%, 1%, or 0.1% of genomes from low, medium and high volume samples, respectively. Coverage was the limiting factor that led to lower fractions at higher volumes (S4 Table). However, we also showed that the mere addition of a barcode led to a reduction in the number of sequenced templates (S1 Text, S4 Fig). In other words, we found that without a barcode we sequence more genomes, but we cannot count how many. There is thus a trade-off between adding a barcode that allows obtaining estimates of template counts, and avoiding the use of barcoding that allows for more template capture. For the HIV and CMV samples we chose the latter approach, since we could use our previous barcoding results as a lower bound on the % of templates sequenced (S3 Table).

**Table 1:** Details of samples sequenced from clinical virus samples.

Virus	#Samples	Sampled tissue	ETI^a^	Region sequenced (NCBI ID)	Average number of viruses sequenced per sample^b^
HIV	9	Plasma	2–5	5’ LTR, Gag, Pol 532–3280 (K03455)	~5,400
RSV	22	Nasal/ throat swabs	<1	G, F, M2, L 4640–14350 (U39661)	~1,700
CMV	12	Amniotic fluid / Urine / saliva	> = 4	UL54 78200–81912 (NC_006273)	~10,000

^a^ Estimated time (weeks) since infection

^b^ Based on barcoded sequences or estimated from viral load and protocol efficiency (S3 Table)

We focused our sequencing efforts mostly on conserved genes, since we expect less diversity and we wanted to test our ability to detect variation that has often been unobserved in the past. Hyper-variable genes such as the HIV-1 envelope have been sequenced extensively using other sequencing approaches [15,16,17], and the presence of high frequency variation is less surprising in such genes. We thus chose the Gag-Pol open reading frame for HIV, the M2 and L open reading frames (encoding for the viral polymerase) for RSV, and the UL54 (also encoding for the viral polymerase) for CMV. In order to allow for comparison, we also sequenced the F and G envelope glycoproteins genes in RSV, and further compared our results to previous sequencing results of the envelope gene in HIV.

**Fig 1:**
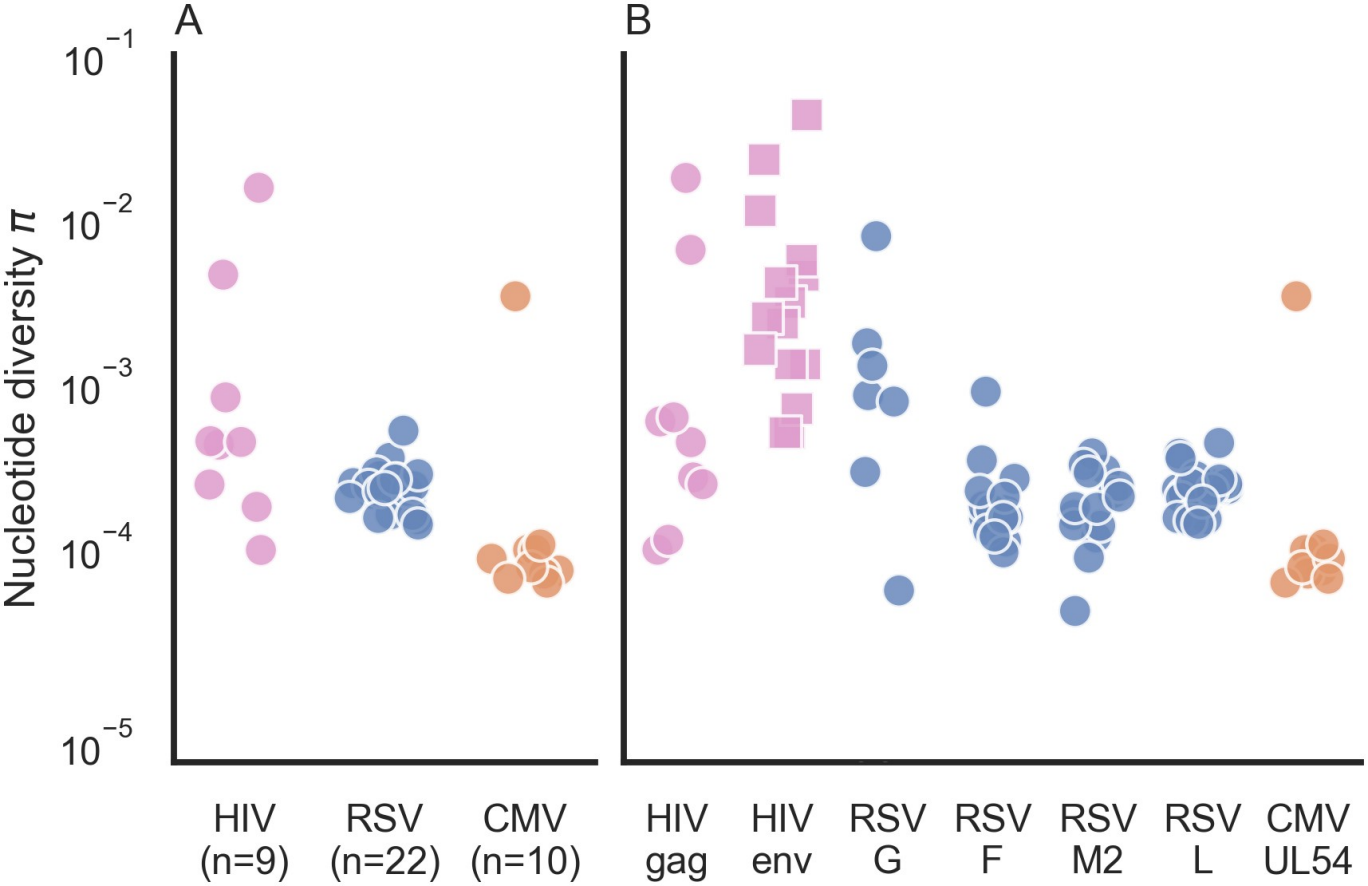
Nucleotide diversity π for acute infections across different virus samples and genes. (A) Each point represents the π diversity of a single sample, across all genes sequenced. Diversity values were calculated using transition mutations only. (B) Gene by gene breakdown of nucleotide π diversity. “gag” represent the gag-pol reading frame. Values for HIV envelope (env) (squares) were taken from previously published data [15].

Each sample underwent population sequencing and variant calling using AccuNGS (see Materials and Methods). We excluded three RSV samples where less than 300 viruses were sequenced. We began by calculating the nucleotide diversity π in each sample based on the transition variants (Materials and Methods). This revealed different distributions of diversity within and between viruses (Fig 1A). In the HIV samples, diversity values spanned several orders of magnitude. On the contrary, RSV samples exhibited very similar intermediate levels of diversity. Similarly, CMV samples usually displayed the lowest diversity with the exception of one sample. We set out to understand what factors drive the differences in diversity among the different samples.

### Mutation and selection

We first considered the two most evident evolutionary causes of differences in diversity: mutation and selection. First, when considering the mutation rate of a virus, the only DNA virus in our data is known to have a lower mutation rate than RNA viruses [18] and indeed displays lower diversity. The two RNA viruses display more diversity than the DNA virus CMV, but the variation in diversity levels is much higher in HIV than in RSV. Of note, the presence of a reverse-transcription step during HIV and RSV sequencing may contribute to some of the observed differences between CMV and the RNA viruses.

We considered whether differences in selection pressure cause the variation in diversity we see among the RNA virus samples. This was unlikely to cause within-virus differences, since we sequenced the same set of genes within each of the virus samples. We did note that the immunogenic envelope proteins in this study (HIV Env and RSV G proteins), often known to be under positive selection [19–21], displayed on average higher diversity than the conserved genes (Fig 1B). This suggests that despite the early stage of infection, some form of immune pressure may already be operating, yet this merits further investigation. However, this could not explain why we saw dramatic differences in diversity in different samples from the same virus when focusing on the same gene (e.g., *gag* in HIV).

### Transmission bottleneck size as a contributor to genetic diversity during acute infections

**Fig 2:**
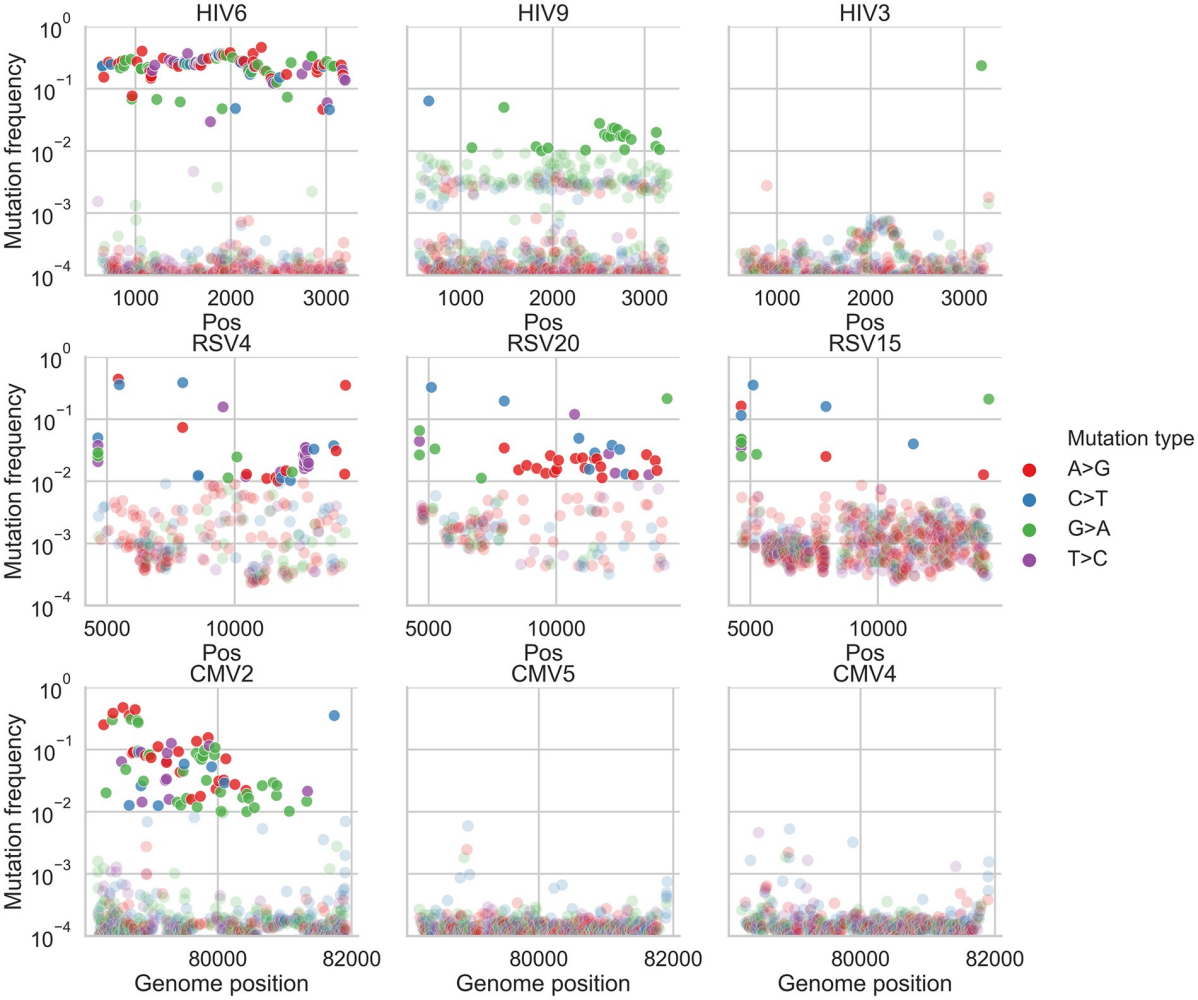
Variant frequency plots in representative samples. Shown are frequencies of transition variants called by AccuNGS, for representative samples from each virus (HIV, top row, RSV, middle row, CMV, bottom row). Variant frequencies lower than 1% are faded. Samples exemplify mixed genotype infections (HIV6, CMV2), mutation biases and presumable hypermutation via host editing (HIV9, RSV samples), and relatively homogenous populations (HIV3, CMV5, CMV4) (see text for details).

It has previously been noted that infections initiated by a few different divergent viruses are characterized by higher genetic diversity [16,22]. Visual inspection of our frequency plots (Fig 2, S5, S6 and S7 Figs) suggested that often variant frequencies were strongly imbalanced, also evident as “bands” of variants at similar frequencies. For example, sample HIV6 (measured π diversity 1.46x10^-2^) contained many variants segregating at a frequency of ~2x10^-1^ yet very few variants segregated at frequencies between 10^−3^ and 10^−1^ (Fig 2). We first considered how likely it is that such a sample would be initiated by only one founder virus/genotype, where all variants begin at a defined frequency of zero. Given a large enough population size and a mutation rate in the order of 10^−5^ mutations/site/day [23], we expect neutral variants that are likely generated over and over almost every day to roughly reach a frequency of 10^−4^–10^−3^ after a few weeks of infection, which is much lower than 10^−1^. Genetic drift or positive selection could drive a few variants to increase in frequency over a short time; however, it seems extremely unlikely that there is such a large set of sites under the exact same regime of positive selection, especially as we had sequenced a gene where positive selection is less prevalent, at least this early in the course of the infection. Thus, it seems quite unlikely that very high diversity samples containing many high frequency variants are founded by one virus genotype, and a more likely explanation is the presence of multiple transmitted/founder viruses.

### Inferring haplotypes and multiple founders

To evaluate the number of founder viruses we require an estimation of the different haplotypes present in a sample, and their abundances. However, reconstruction of virus haplotypes from short reads and from one time-point is a longstanding problem [24]. This is due to two conflicting features of viral population sequencing data: on the one hand, the data is often too homogenous. In other words, most reads are identical or almost identical to the consensus, and there may not be enough variants on one read that allow “linking” it with another read. On the other hand, the mutation rates of viruses may scale with sequencing error rates, throwing off most commonly used haplotype reconstruction methods.

**Fig 3:**
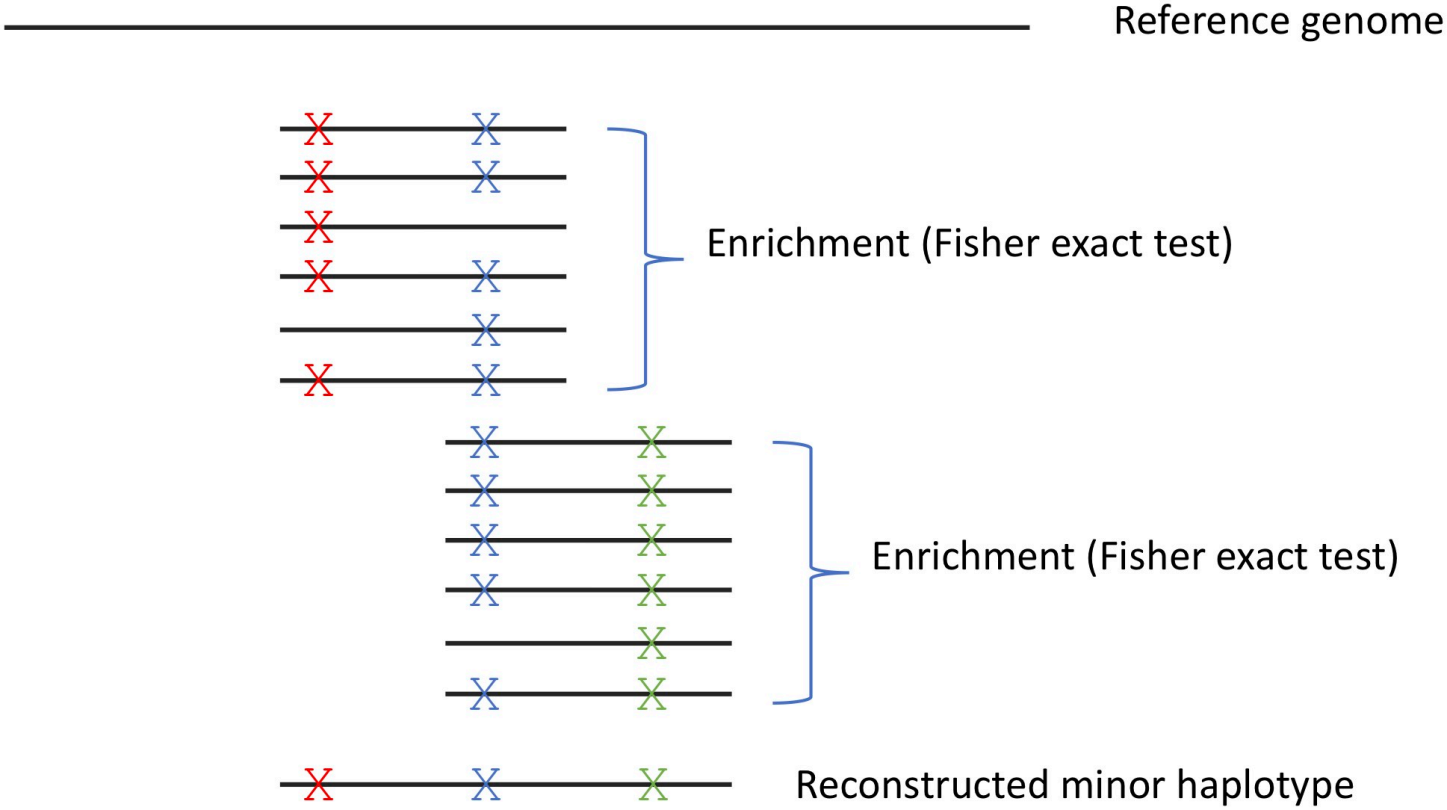
Illustration of method for haplotype reconstruction. The method searches for enrichment of pairs of mutations on the same read, and concatenation of enriched reads that share a mutation into a reconstructed minor haplotype. Notably, the concatenation approach is suitable for populations with limited diversity, as is the case in acute infections; in highly diverse populations, many haplotypes may share the “blue” mutation illustrated in the figure.

We thus set out to develop a new approach for inferring viral haplotypes. Instead of attempting to reconstruct the entire haplotype, we mainly focused on inferring if more than one haplotype is present in a sample. Our approach is based on looking for statistical enrichment for two variants being present on the same read as opposed to each variant on its own, and then linking these reads one with another based on shared variants (Materials and Methods, Fig 3) (see for example [25,26,27]). Notably, this approach is valid only for acute infections, where initial genetic diversity is low. Otherwise, different reads may share mutations since these mutations occurred independently in the course of a long infection, throwing our method off. Moreover, the method will only detect haplotypes that are quite divergent from each other, since it searches for two mutations per every 250 bases, the size of a sequencing read.

Our haplotype reconstruction approach also led us to realize one of the combined strengths and pitfalls of ultra-deep sequencing: we were able to initially detect minute contaminations (a few hundred out of millions of reads) from one sample into another, which we were then able to computationally filter out. When interrogating the source of contamination, we pinpointed the most likely reason to be barcode contamination (S1 Text). On the other hand, we were reassured that the haplotype reconstruction tool of AccuNGS allowed for the clear-cut detection and evaluation of a contamination, which we believe is very important to capture.

**Fig 4:**
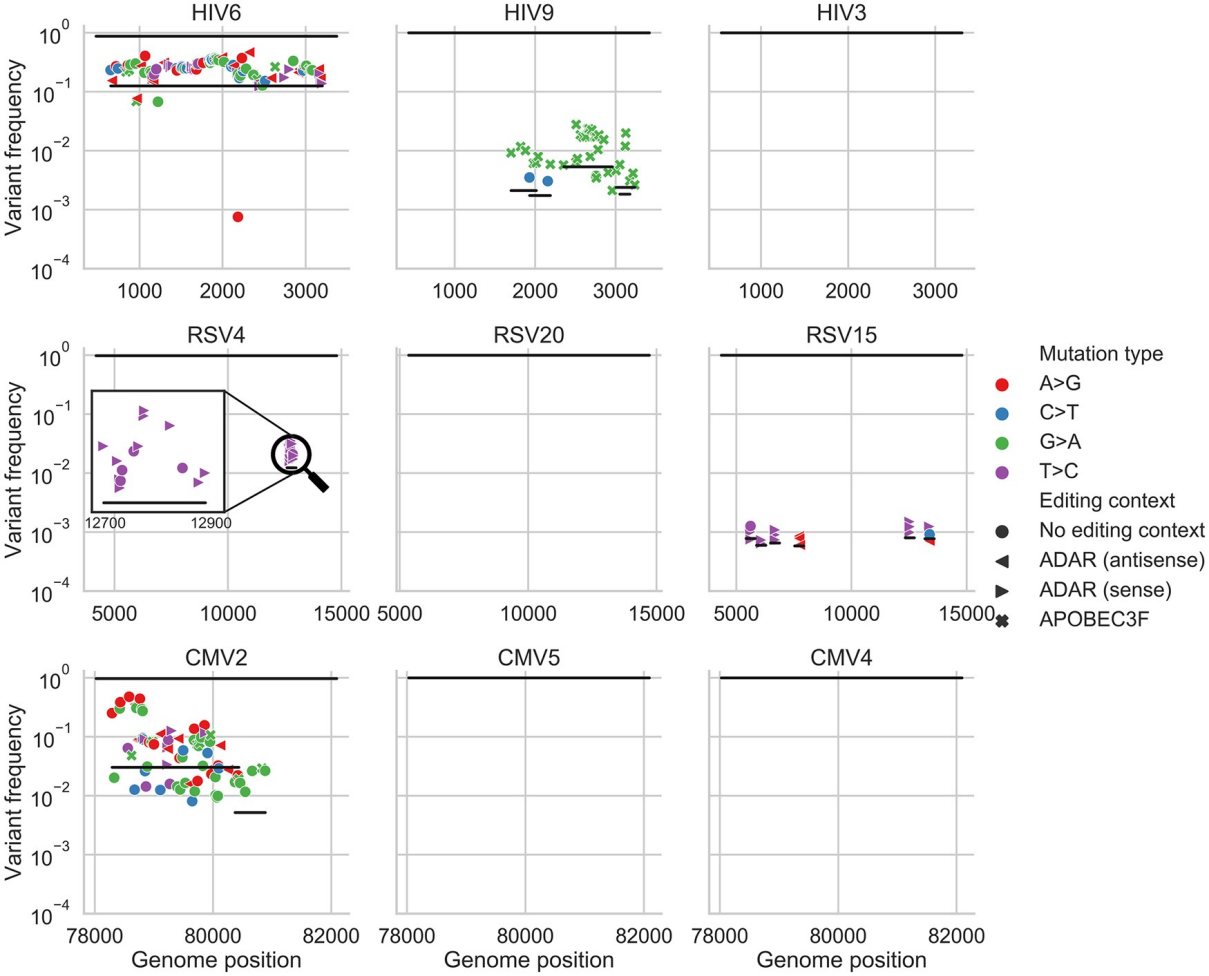
Haplotype reconstruction based on co-occurrence of variants on the same reads. Shown are inferred haplotypes (lines) based on consecutive significant associations of pairs of variants (shapes) one to another on the same read. The uppermost line in each panel represents the consensus sequence, which by definition is the major haplotype in each sample. Both HIV6 and CMV2 samples show strong evidence of an additional haplotype, which is likely a second founder genotype. Sample HIV9 shows evidence of G>A hyper-mutation in the context of APOBEC3 editing, samples RSV4 and RSV15 show evidence of T>C or A>G hyper-mutation in the context of ADAR editing in regions spanning a few hundred bases. The hyper-mutated region in RSV4 sample is magnified for clarity. “Empty” panels signify what are likely single haplotype infections, with no evidence of hyper-mutation.

We next applied our haplotype inference flow to all the filtered samples, and found that all of the high diversity samples (diversity >10^−3^, two HIV samples and one CMV sample, Fig 1A) exhibited strong evidence for containing two or more divergent haplotypes (S5, S6 and S7 Figs). Two examples are shown in Figs 2 and 4: HIV sample 6 has a “band” of variant frequencies around 2x10^-1^ (Fig 2), and indeed most of these variants can be linked to each other in this sample (Fig 4). CMV sample 2 has a wide “band” of variant frequencies between 10^−2^ and 5x10^-1^ (Fig 4), which were also mostly found to be linked, and likely represent a founder haplotype and the associated variants that were created on the background of this haplotype (Fig 4). In general, we found no evidence for two or more haplotypes in the less diverse samples, except for the most diverse RSV sample (highest blue circle in Fig 1A) that also showed limited evidence of a low frequency haplotype (S6 Fig) (see discussion).

### Short hyper-mutated genomic stretches

One well-known phenomenon of HIV infections is the potential of host APOBEC3 (A3) proteins to induce hyper-editing on the negative strand of nascent HIV DNA during reverse transcription, resulting in an excess of G>A mutations in regions of the RNA genome [28,29]. This hyper-mutation strategy is thought to lead to DVGs that are unable to replicate. However, HIV encodes a *vif* gene that counteracts A3 proteins, and thus most HIV viruses sequenced from blood samples show only minor evidence for A3 activity [30]. Similarly, the family of human ADAR proteins have also been shown to induce A>I mutations (read as A>G mutations) in a variety of viruses [31]. We set out to test if we detect signals of hyper-editing in our samples. In particular we sought to find stretches of hyper-mutations using our haplotype reconstruction approach in order to evaluate whether hyper-editing contributes to the observed genetic diversity, and to what extent.

Of all 43 samples from the three different viruses, only one HIV sample (HIV9, Fig 2) displayed strong evidence for G>A hyper-mutation, evident in Fig 2 as multiple green dots above and below a frequency of 1%. In this sample, editing seemed to be widespread, with multiple distinct and overlapping hyper-mutated haplotypes (Fig 4). Hyper G>A mutations were enriched in the context of GpA which is the APOBEC3D/F/H favored editing context but not of the canonical APOBEC3G [32,33]. Most variants on these hyper-mutated stretches were missense variants; some of these stretches contained variants that lead to premature stop codons which are presumably lethal for the virus (S5 Fig). The maximum frequency of such variants in the sample was roughly 2x10^-2^. To test whether this occurs due to an inactive *vif* gene, we sequenced this gene in this sample using AccuNGS. We found no support for this hypothesis since the consensus sequence of this gene was intact, but we once again noticed a relatively high level of G>A mutations in the *vif* gene itself (not shown), and also noted that the pattern of hypermutation was repeated in three independent sequencing replicates of this sample (S8 Fig). Notably, this sample had the highest viral load among all HIV samples sequenced in this study (S3 Table).

Out of 22 RSV samples, 11 (50%) exhibited evidence of ADAR-mediated hyper-edited genomes, manifested as at least three ADAR-associated mutations on the same haplotype [34]. When observed, ADAR-like linked variants were present at frequencies varying between ~10^−3^ and ~10^−2^, which by far exceed the mutation rate of any known virus. Out of 26 ADAR-like hyper-edited haplotypes, 23 of them were on the negative strand and only 3 were on the positive strand, in line with previous studies demonstrating that most ADAR-like mutations are acquired on the negative strand of (-)ssRNA viruses e.g., [34]. Most of the ADAR-like variants on these stretches were missense variants, suggesting they have a detrimental effect on the virus (S6 Fig).

None of the CMV samples exhibited any A3, ADAR, or other pattern of hyper-mutation, suggesting that these hyper-mutating enzymes do not act on CMV samples, at least not for the gene sequenced here, or at the level of detection of AccuNGS (but see [35]).

## Discussion

Next generation sequencing has become a key tool for the discovery and investigation of pathogen dynamics. We begin here by describing AccuNGS, a simple and rapid pipeline that allows characterizing the genetic diversity in low-biomass clinical RNA and DNA virus samples. We also would like to outline the pitfalls of AccuNGS, and of sequencing approaches in general, which always essentially report results from a sample of genomes. As shown herein, this sampling may create strong biases in reported mutation frequencies. However, we also show that is worthwhile focusing on measures that take into account multiple mutations (π diversity, or inferred haplotypes). Moreover, we suggest that the depth obtained by AccuNGS (number of templates sequenced) has allowed us to obtain many of the conclusions reached herein, especially the ability to detect low frequency haplotypes.

We used AccuNGS to characterize HIV-1, RSV, and CMV diversity. The HIV-1 samples were from early stages of infection, typically 2–5 weeks post infection, based on serology testing. The RSV samples were taken from children hospitalized due to respiratory problems, about 3–5 days post infection [36], while the CMV samples taken from amniotic fluid or newborn urine and saliva are several weeks post infection.

Our results suggest that a prominent factor in determining the intra-host genetic diversity of a sample during acute infection is the number of diverse genotypes present in a sample, and we find that the samples with the highest levels of diversity always show evidence for the existence of multiple genotypes. We suggest that these different genotypes reflect different founders/transmitted viruses, although definitive evidence for this would be from donor-recipient pairs. Accordingly, we found evidence for multiple founder infections in two of nine HIV-1 samples, in line with previous reports [16]. A debate has arisen regarding the diversity in CMV samples, where one study has claimed that diversity in this DNA virus is comparable to diversity in RNA viruses [37], and others suggest that diversity is low in single founder infections and is elevated only when multiple founders/genotypes initiate the infection [22]. Our results strongly support the latter hypothesis.

For RSV samples, apart from possibly a single sample we found no evidence for multiple haplotypes in the samples, which is somewhat surprising given that this is an airborne virus that reaches high titers. Previous work has suggested that the number of founders in RSV infections is 25±35 [38]. Notably, these values were obtained for adults experimentally inoculated with RSV, whereas our study represent natural infection of infants. However, another explanation for this discrepancy is that our data does not allow us to detect infection with multiple founders when they share very similar genotypes, since our haplotype reconstruction method relies on detecting short reads that share two or more mutations. Given the very short duration of RSV infection, it is possible that relatively little genetic diversity is created *de novo*, and hence very little genetic diversity is transmitted. In other words, an infection may be initiated by several very genetically similar founder genotypes, but we would not detect it. On the other hand, CMV and HIV create longer infections, and the potential to generate and transmit more diverse genotypes within a single carrier is higher.

Our results enabled pinpointing the activity of viral hyper-editing by host enzymes, namely APOBEC3 enzymes and ADAR. The latter was particularly prominent in the RSV infections, where we found distinct clusters of mutations matching ADAR context. Surprisingly, the frequency of these clustered mutations was often relatively quite high, as discussed above. There is a debate today surrounding the role of ADAR in viral infections: in some case it was found to be pro-viral whereas in other cases it has been shown to be anti-viral. Pro-viral activity may be plausible when considering that it has been found that ADAR protects cellular transcripts from being detected by intracellular innate immune response [39,40].

Is it possible that the ADAR signatures we find represent edited viral genomes that escape innate immunity? If so, this would mean these are not DVGs but rather haplotypes with a selective advantage. We consider this unlikely: many of the ADAR-like mutations we find are non-synonymous, with often 5–10 such mutations found in a short region. It is highly improbable that so many mutations would yield a “viable” genome, and we hence conclude that ADAR-like hyper-editing yields DVGs. We find that the most likely explanation for this phenomenon is that cellular co-infection is very common in RSV, which may be promoted by the syncytia that RSV creates, allowing for complementation of these DVGs. We suggest that RSV infections may occur in a relatively dense site, which allows for so many co-infections, and for the propagation of DVGs. We suggest that the use of AccuNGS can allow an in-depth understanding of DVGs and genetic variation in clinical samples, allowing a better and more detailed understanding of the processes that govern evolution.

## Materials and methods

### Ethics statement

The study was approved by the local institutional review boards of Tel-Aviv University, Sheba Medical Center (approval number SMC 4631–17 for HIV and SMC 5653–18 for RSV), and Haddasah Medical Center (approval number HMO-063911 for CMV). All samples were retrospective and obtained from leftover material used for routine diagnosis. Samples were fully anonymized. Hospital approvals included exemption from informed consent under these circumstances.

### Reagents and kits

Unless stated otherwise, all the described reactions in this paper were carried with the described products according to the manufacturer’s instructions: gel purifications were performed using Wizard SV Gel and PCR Clean-Up System (Promega, Madison, WI, USA); beads purifications were performed using AMPure XP beads (Beckman Coulter, Brea, CA, USA); concentrations were determined using Qubit fluorometer (Thermo Fisher Scientific, Waltham, MA, USA); reverse transcription (RT) reactions were performed using SuperScript III or IV Reverse Transcriptase (Thermo Fisher Scientific); polymerase chain reactions (PCR) were made using Platinum SuperFi high-fidelity DNA Polymerase (Thermo Fisher Scientific) or Q5 high-fidelity DNA Polymerase (New England Biolabs (NEB), Ipswich, MA, USA).

### Generation of amplicons from HIV-1 clinical samples

#### Clinical HIV-1 samples

Plasma samples from nine recently diagnosed HIV-1 patients with viral loads of 5x10^5^-1x10^7^ cp/ml were provided by the National HIV Reference Laboratory, Chaim Sheba Medical Center, Ramat-Gan, Israel (S3 Table). HIV-1 viral loads were determined and RNA extracted from 0.5 mL as described above. From each sample a maximum of ~300,000 HIV-1 copies were reverse transcribed using random hexamer priming.

#### Generation of Gag-Pol amplicons

The cDNA of the 9 HIV-1 clinical samples and the HIV-1 control sample were used to generate amplicons. To remove excess primers, the resulting cDNA was beads purified (0.5X ratio) and eluted with 30μl nuclease-free water. Fifteen microliters of each sample were then used for PCR amplification using SuperFi DNA polymerase. To amplify ~2500 bp spanning entire Gag and part of Pol HIV-1 regions (HXB2 coordinates 524–3249), the following primers were used: GAG FW 5'CTC AAT AAA GCT TGC CTT GAG TGC and RT gene RV 5'ACT GTC CAT TTA TCA GGA TGG AG, and the following PCR program: initial denaturation for 3min at 98°C, followed by 40 cycles of denaturation for 20sec at 98°C, annealing for 30sec at 62°C and extension for 2.5min at 72°C, and final extension for 5min at 72°C. The amplicons were gel purified and their concentration was determined. The purified products were further used for library construction.

#### Generation of Gag amplicon with primer-ID from HIV9 sample

A primer specific to the entire Gag gene of HIV-1 (HXB2 position 2347) was designed with a 15 N-bases unique barcode followed by a linker sequence for subsequent PCR, Gag ID RT 5’TAC CCA TAC GAT GTT CCA GAT TAC GNN NNN NNN NNN NNN NAC TGT ATC ATC TGC TCC TG TRT CT. Based on the measured viral load and sample concentration, 4 μl (containing roughly 300,000 HIV-1 copies) were taken for reverse transcription reaction. Reverse transcription was performed using SuperScript IV RT with the following adjustments: (1) In order to maximize the primer annealing to the viral RNA, the sample was allowed to cool down gradually from 65°C to room temperature for 10 minutes before it was transferred to ice for 2min; And (2) The reaction was incubated for 30min at 55°C to increase the overall reaction yield. To remove excess primers, the resulting cDNA was beads purified (0.5X ratio) and eluted with 35μl nuclease-free water. To avoid loss of barcoded primers (“primer-ID”s) due to coverage drop at the ends of a read as a result of the NexteraXT tagmentation process (see "Miseq/Nextseq Libraries construction"), the PCR forward primer was designed with a 60bp overhang so the barcode is far from the end of the read. The primers used for amplification were Gag ID FW 5’CTC AAT AAA GCT TGC CTT GAG TGC and Gag ID RV 5’AAG CGA GGA GCT GTT CAC TGC CAT CCT GGT CGA GCT ACC CAT ACG ATG TTC CAG ATT ACG. PCR amplification was accomplished using SuperFi DNA polymerase in a 50μl reaction with 33.5μl of the purified cDNA as input using the following conditions: initial denaturation for 3min at 98°C, followed by 40 cycles of denaturation for 20sec at 98°C, annealing for 30sec at 60°C and extension for 1min at 72°C, and final extension for 2min at 72°C. The Gag amplicon was gel purified and the concentration was determined. The purified product was further used for library construction.

#### Generation of a Vif amplicon from HIV9 sample

One and a half microliters from clinical sample HIV9 were reverse transcribed using SuperScript IV RT and random hexamer priming. Five microliters of the purified RT reaction were used to set-up a PCR reaction with SuperFi DNA polymerase to amplify ~600 bp region spanning HIV-1 Vif gene using primers vif FW 5'AGG GAT TAT GGA AAA CAG ATG GCA GGT and vif RV 5'CTT AAG CTC CTC TAA AAG CTC TAG TG, and the following program: initial denaturation for 3min at 98°C, followed by 40 cycles of denaturation for 20sec at 98°C, annealing for 30sec at 60°C and extension for 30min at 72°C, and final extension for 5min at 72°C. The amplicon was gel purified and the concentration was determined. The purified product was further used for library construction.

### Generation of amplicons from RSV clinical samples

#### Clinical RSV samples

Nasopharyngeal samples of 25 patients hospitalized at Chaim Sheba Medical Center (S3 Table) were collected into Virocult liquid viral transport medium (LVTM) (Medical Wire & Equipment Co, Wiltshire, United Kingdom) and stored at -70°C. Five hundred microliters of each sample were extracted and purified using easyMAG according to the manufacturer’s instructions. A primer specific to the glycoprotein protein G was designed with a 15 N-bases unique barcode followed by a linker sequence for subsequent PCR, RSV G RT 5’TAC CCA TAC GAT GTT CCA GAT TAC GNN NNN NNN NNN NNN NGC AAA TGC AAM CAT GTC CAA AA. Eight microliters of each sample were reverse transcribed as described in “Generation of Gag amplicon with primer-ID from HIV9” section.

#### RSV control sample

In the absence of an RSV plasmid, we used human rhinovirus (RV) plasmid (a kind gift by Ann Palmenberg (University of Wisconsin-Madison, WI, USA)) to generate a homogeneous control that was run on the same Nextseq run as the RSV samples, similar to the described above. In vitro transcribed RNA underwent RT using SuperScript IV with the following primer: RV14 5' TAC GCA TAC GAT GTT CCA GAN NNN NNN NNN NNN NNN NNN NAT AAA CTC CTA CTT CTA CTC AAA TTA AGT GTC. PCR amplification using Q5 DNA polymerase with the following primers was performed: p3.26 FW 5' TTA AAA CAG CGG ATG GGT ATC CCA C and p3.26 RV 5'ATG GTG AGC AAG GGC GAG GAG CTG TTC ACC GGG GTG GTG CTA CGC ATA CGA TGT TCC AGA.

#### Generation of a glycoprotein-fusion protein amplicon and polymerase amplicons

PCR amplification was accomplished using Q5 DNA polymerase in 50μl reactions with 15μl of the purified cDNA as input. The following conditions were used for the glycoprotein-fusion protein amplicon: initial denaturation for 3min at 98°C, followed by 40 cycles of denaturation for 20sec at 98°C, annealing for 30sec at 58°C and extension for 3.5min at 72°C, and final extension for 5min at 72°C, using the following primers: Extension FW 5’AAG CGA GGA GCT GTT CAC TGC CAT CCT GGT CGA GCT ACC CAT ACG ATG TTC CAG ATT ACG and RSV G and F RV 5’TGA CAG TAT TGT ACA CTC TTA. For the polymerase amplicon, the following conditions were used: initial denaturation for 3min at 98°C, followed by 40 cycles of denaturation for 20sec at 98°C, annealing for 30sec at 60°C and extension for 8min at 72°C, and final extension for 5min at 72°C, using the following primers: RSV L FW 5’GGA CAA AAT GGA TCC CAT TAT T and RSV L RV 5’GAA CAG TAC TTG CAY TTT CTT AC. The amplicons were beads purified and joint together at equal amounts. Concentration was determined, and the product was further used for NextSeq library construction.

### Generation of a UL54 amplicon from CMV clinical samples

Clinical DNA samples of recently infected patients (see S3 Table) were obtained and purified as described previously [35]. Since CMV is a DNA virus, no reverse transcription step was needed. To generate a homogeneous control sample, the UL54 gene from TB40/E strain was cloned onto a pGEM-t plasmid as described previously [35]. The samples were diluted to 30,000 copies per PCR amplification reaction, which was set-up using the Q5 DNA polymerase. The primers used to amplify the UL54 gene were UL54 FW 5'TCA ACA GCA TTC GTG CGC CTT and UL54 RV 5'ATG TTT TTC AAC CCG TAT CTG AGC GGC, and the following PCR protocol was executed: initial denaturation for 3min at 98C, followed by 38 cycles of denaturation for 20sec at 98C, annealing for 20sec at 65C and extension for 3min at 72C, and final extension for 5min at 72C. The amplicons were beads purified and their concentrations were determined. The purified products were further used for MiSeq library construction with the following change, 0.875ng of DNA were used as input for tagmentation instead of 0.85ng.

### MiSeq/Nextseq Libraries construction

PCR fragmentation and indexing of samples for sequencing was performed using the Nextera XT DNA Library Prep Kit (Illumina, San Diego, CA, USA) with the following adjustments to the manufacturer instructions; (1) In order to get a short insert size of ~250bp, 0.85 ng of input DNA was used for tagmentation; (2) No neutralization of the tagmentation buffer was done, as described previously [41]; (3) For library amplification of the tagmented DNA, the Nextera XT DNA library prep PCR reagents were replaced with high-fidelity DNA polymerase reagents (the same DNA polymerase that was used for the amplicon generation). The PCR reaction (50μl total) was set as depicted. Directly to the tagmented DNA, index 1 (i5, illumina, 5μl), index 2 (i7, illumina, 5μl), buffer (10μl), high-fidelity DNA polymerase (0.5μl), dNTPs (10mM, 1μl) and nuclease-free water (8.5μl) were added; (4) Amplification was performed with annealing temperature set to 63°C instead of 55°C, as introduced previously [41] and final extension for 2min; (5) Post-amplification clean-up was achieved using AMPure XP beads in a double size-selection manner [42], to remove both too large and too small fragments in order to maximize the fraction of fully overlapping read pairs. For the first size-selection, 32.5μl of beads (0.65X ratio) were added to bind the large fragments. These beads were separated and discarded. For the second-size selection, 10μl of beads (0.2X ratio) were added to the supernatant to allow binding of intermediate fragments, and the supernatant containing the small fragments was discarded. The intermediate fragments were eluted and their size was determined using a high-sensitivity DNA tape in Tapestation 4200 (Agilent, Santa Clara, CA, USA). A mean size of ~370bp, corresponding to the desired insert size of ~250bp, was achieved; And (6) Normalization and pooling was performed manually.

NextSeq: The longest NextSeq read length is 150bp, we hence selected for a shorter insert size of 270bp, compared to the desired 370bp insert size for the MiSeq platform. The first size selection of the post-NexteraXT amplification cleanup was performed using 42.5μl of AMPure XP beads (0.85X ratio) [42].

### AccuNGS protocol evaluation

The AccuNGS protocol was evaluated using HIV-1 DNA plasmid [43]. Our underlying assumption was that this DNA starting material is homogenous with respect to the theoretical error rate we calculated. This assumption was based on the fact that we used low-copy plasmids that were grown in *Escherichia coli*, and only a single colony was subsequently sequenced. The mutation rate of *E*. *coli* is in the order of 1x10^-10^ errors/base/replication [44], and accordingly, error rates in the purified plasmids are expected to be much lower than the expected protocol mean error of ~10^−5^, which is based on error rates of the polymerases of the protocol and the use of overlapping reads with Q30.

#### Preparation of plasmids

In order to maintain the plasmid stock as homogenous as possible, plasmids were transformed to a chemically competent bacteria cells [DH5alpha (BioLab, Israel) or TG1 [A kind gift by Itai Benhar (Tel Aviv University, Tel Aviv, Israel)]] using a standard heat-shock protocol. Based on the fact that *E*. *coli* doubling time is 20 minutes in average using rich growing medium [45], a single colony was selected and grown to a maximum of 100 generations. Plasmids were column purified using HiYield Plasmid Mini Kit (RBC Bioscience, New Taipei City, Taiwan) and stored at -20°C until use.

#### Construction of a baseline control DNA amplicon

A baseline control amplicon was based on clonal amplification and sequencing of the pLAI.2 plasmid, which contains a full-length HIV-1_LAI_ proviral clone [43] (obtained through the NIH AIDS Reagent Program, Division of AIDS, NIAID, NIH: pLAI.2 from Dr. Keith Peden, courtesy of the MRC AIDS Directed Program). The Integrase region of pLAI.2 was amplified using primers: KLV70–5’TTC RGG ATY AGA AGT AAA YAT AGT AAC AG and KLV84–5’TCC TGT ATG CAR ACC CCA ATA TG [46]. PCR amplification was conducted using SuperFi DNA Polymerase in a 50μl reaction using 20–40 ng of the plasmid as input. Amplification in a thermal cycler was performed as follows: initial denaturation for 3min at 98°C, followed by 40 cycles of denaturation for 20sec at 98°C, annealing for 30sec at 60°C and extension for 1min at 72°C, and final extension for 2min at 72°C. In parallel, an alternative PCR reaction was up using Q5 DNA Polymerase. The Integrase amplicon was gel purified and concentration was determined. The purified product was further used for library construction.

#### Generation of synthetic populations mimicking clinical samples, based on HIV-1 RNA dilutions

Fifty nanograms of the plasmids pLAI.2 and pNL4-3 entered a PCR reaction in order to create homogenous RNA using a primer containing the sequence of the T7 RNA polymerase promotor 5’TAA TAC GAC TCA CTA TAG CTG GGA GCT CTC TGG CTA AC and the primer pLAI 5761–5782 5’GAG ACT CCC TGA CCC AGA TGC C using Q5 DNA polymerase and the following PCR program: initial denaturation for 3min at 98°C, followed by 40 cycles of denaturation for 10 sec at 98°C, annealing for 30sec at 65°C and extension for 3min at 72°C, and final extension for 5min at 72°C. Twelve microliters from PCR reaction were carried to a 30ul in-vitro transcription reaction using HiScribe T7 High Yield RNA Synthesis Kit (NEB) according to the manufacturer’s instruction. The reactions were carried on to DNaseI treatment (NEB) in order to clean up any residual DNA. Finally, the reactions were purified using RNA Clean & Concentrator by Zymo according to manufacturer’s instructions. RNA that was generated from pLAI.2 was diluted into pNL4-3 RNA’s to the following concentrations based on QuiBit measurements: 1 (pLAI.2 copies):10,000 (pNL4-3 copies), 1:5,000, 1:2,000, 1:1,000 and 1:100. The dilutions were performed in three independent biological replicates (A, B and C) with three different volumes of initial copies per sample: high (1M copies), medium (100,000 copies) and low (10,000 copies) based on QuBit measurements (weight to copies conversion). A total of 45 different samples were created.

#### Sequencing

Sequencing of all synthetic samples, the HIV-1, and CMV samples was performed on the Illumina MiSeq platform using MiSeq Reagent Kit v2 (500-cycles, equal to 250x2 paired-end reads) (Illumina). Sequencing of the RSV-1 samples and a dedicated synthetic sample was performed on the Illumina NextSeq 500 platform using NextSeq 500/550 High Output Kit (300-cycles, equal to 150x2 paired-end reads) (Illumina).

### Barcode serial dilution test

The pLAI.2 plasmid was used to generate an RNA pool. Five micrograms of this plasmid were linearized using SalI (NEB) and beads purified (0.5X ratio). T7 polymerase promotor was added to the linearized plasmid using T7 extension FW 5’TAA TAC GAC TCA CTA TAG CTG GGA GCT CTC TGG CTA AC and the RV 5’GAG ACT CCC TGA CCC AGA TGC C in a PCR reaction using Q5 DNA polymerase with the following program: initial denaturation for 3min at 98°C, followed by 40 cycles of denaturation for 10sec at 98°C, annealing for 10sec at 65°C and extension for 3min at 72°C, and final extension for 5min at 72°C. Four microliters of the reaction was in-vitro transcribed using T7 RNA polymerase according to the manufacturer’s instructions. The transcribed RNA was beads purified (0.5X ratio). The purified RNA was serially diluted and for each dilution two reactions were set-up: a primer-ID reaction (as described in the section “Generation of Gag amplicon with primer-ID from HIV-1”) and a random hexamer based RT reaction (as described in the section “Construction of RNA control amplicons”). In order to compare these reactions, for the PCR amplification of the random hexamer based RT reaction, we used the following primers: GAG FW 5’CTC AAT AAA GCT TGC CTT GAG TGC and RTgene RV 5'ACT GTA TCA TCT GCT CCT GTA TCT corresponding to the primer-ID reaction primers without a barcode. The same PCR program was used for both reactions. The PCR reactions were gel purified and concentration was measured.

### Reads processing and base calling

The paired-end reads from each control library were aligned against the reference sequence of that control using an in-house script that relies on BLAST command-line tool [47–49]. The paired-end reads from the clinical samples were aligned against: HIV-1 subtype B HXB2 reference sequence (GenBank accession number K03455.1), RSV reference sample (GenBank accession number U39661), CMV reference sample Merlin (GenBank accession number NC_006273), and then realigned against the consensus sequence obtained for each sample. Bases were called using an in-house script only if the forward and reverse reads agreed and their average Q-score was above an input threshold (30 or 38). At each position, for each alternative base, we calculate mutation frequencies by dividing the number of reads bearing the mutation by loci coverage. In order to analyze the errors in the sequencing process we used Python 3.7.3 (Anaconda distribution) with the following packages: pandas 0.25.1 [50], matplotlib 3.1.0 [51], seaborn 0.9.0 [52], numpy 1.16.3 [53,54] and scipy 1.2.1 [55].

### Diversity calculation

Transition nucleotide diversity π was calculated per sample using positions with at least 5,000x coverage, using the formulas described in [12], but excluding transversion variants.

### Haplotype inference

To infer potential haplotypes, we used a two-step process, illustrated schematically in Fig 3. First, we identify all pairs of non-consensus variants (the most common minor variant at each site) that are statistically enriched when present on the same reads. Next, we attempt to "link" multiple pairs into a longer stretch based on a shared mutation present in two different pairs of variants. In order to find statistically enriched pairs, we consider all sites that may be linked on the same reads (up to 250 bases, which is the maximal length of an Illumina read). For each pair of loci, we create a contingency table for the appearance of each variant alone, the two variants together and no variant at all. We then use a one-tailed Fisher exact test to obtain a p-value for the pair, and considered only p-values lower than 10^−15^, to account of multiple testing. From this contingency table we also extract the frequency at which the two variants co-occur. We repeat the process for all possible pairs of loci. This results in many short haplotype stretches of 250 bases spanning two loci each. We then perform "linking" of pairs of loci that have (1) at least one shared position and (2) a similar frequency of co-occurrence, defined here as up to an order of magnitude in difference. Such linked loci form a longer stretch and its frequency is calculated as the mean frequency of its components, i.e., the average frequency of all individual pairs added to this stretch so far. For each sample, we iteratively attempt to concatenate all pairs of loci, starting from the highest frequency pair to the least common pair, until no pairs can further merge.

## Supporting information

S1 Text AccuNGS validation. (DOCX)

S1 Table Error rates for AccuNGS on high volume DNA. Rates shown were calculated based on a Q30 score cutoff.
(XLSX)

S2 Table SuperScript III median error rates estimations on high volume RNA. (XLSX)

S3 Table Summary of all clinical samples sequenced. (XLSX)

S4 Table Summary of RNA control dilution experiments. Shown are the number of input genomes and the number of barcodes that correspond to number of sequenced genomes. n/a corresponds to a failed sequencing run. Coverage was aimed to be uniformly around 30,000 for all samples. For low volume samples, around 10% of genomes were sequenced, for medium volume about 1%, and for high volume samples around 0.1%. This suggests that coverage is the limiting factor in the experiment.
(XLSX)

S1 Fig Mean background error rates of different sequencing protocols at the DNA level. (A) AccuNGS dramatically reduces errors present in standard sequencing protocols by almost two orders of magnitude. For standard sequencing control, a standard homogeneous pLAI.2 control was taken from (Moscona, et al. 2017), and mutations were called without accounting for overlapping paired reads, while considering positions to analysis only if sequenced to at least 2,000x depth. PCR errors in AccuNGS are negligible (in average) based on the comparison of a PCR and PCR-free sample. Higher rates of G>T and C>A are likely indicative of oxidative stress. Error bars represent 95% confidence intervals around estimated mean values using 1,000 bootstrap repeats. (B) The effect of increasing the Q-score filtering threshold on AccuNGS error rates, presented for each type of transition error. A>G and T>C transitions show the most dramatic effect when increasing the Q-score filtering threshold. (C) Distributions of process errors potentially associated with oxidative damage (G:C>T:A). Notably in the In Vitro RNA sample, the C>A errors pattern is different from the pattern observed in other samples due to the single-stranded origin of this sample. Boxplots of errors per type of base changes are shown. Raw read bases were filtered when their average Q-score was less than Q30. (D) Comparison of pi diversity estimates using a standard sequencing approach versus AccuNGS. Substantial differences in pi diversity can be observed on the one biological sample (HIV1) that was sequenced with both methods, and on the pLAI plasmid sequenced with both methods. Also shown are technical replicates of sample HIV9 (see also S8 Fig). **p<0.01; ***p<0.001; ****p<0.0001.
(TIF)

S2 Fig Variant transition frequencies of synthetic RNA control samples. Three different volumes are presented, corresponding to the rows of the table: low viral load (total of 10,000 copies, label = L), medium viral load (total of 100,000 copies, label = M), and high viral load (total of 1M copies, label = H) (replicate B is shown). The expected frequency of the spiked-in minor haplotype/strain is shown at the top of each box. Blue variants were called from the dominant strain used (and hence false positives), and orange variants represent variants that distinguish the dominant and minor strain used (and hence true positives). The mean error rate based on the blue variants was around 5X10-4. However, the variance of errors was higher the lower the volume of the sample, leading to more high frequency errors in the lower volumes. The sequencing run for volume H and dilution 0.01 failed and hence is empty. This experiment was performed in three biological replicates (S4 Table); in this figure only replica B in shown.
(TIF)

S3 Fig Accuracy and reproducibility tested on three serial dilution experiments of the synthetically created RNA populations. Details of the experiment and figure details as in S2 Fig. (A) Nucleotide diversity π measured on non-spiked-in variant positions shows a stable value around 5X10-4, that is independent of the number of input templates, and independent of the second spiked-in haplotype and its frequency. (B) Inferred minor haplotypes. Each type of line (solid, dashed, dotted) corresponds to a different independent biological replicate of the experiment. The higher the input volume, the more low- frequency haplotypes are captured. Haplotypes with multiple G>T/C>A/C>T variants removed. False positives were composed of at most three linked variants. (C) Inference of haplotypes on technical replicates (resequencing) of replicate C (circles, first technical replica; crosses, second replica). We noted X3 lower coverage in the second technical replicate, validated by less barcodes, leading to less inference of the spiked-in variant (Table S5). (D) Scatter-plot of mutation frequencies of the two technical replicates. Blue: false positives (errors), orange: true positives (true variants of spiked-in haplotypes). Reproducibility of true positives is demonstrated in C & D.
(TIF)

S4 Fig Adding a barcode leads to reduced yield. PCR results of serially diluted samples run with RT that includes a barcode (top) compared to RT without a barcode (bottom). The row corresponding to the estimated produced size (1935 bp with a barcode and 1860 bp without a barcode) is boxed. Estimated number of templates following dilution is shown on the right.
(TIF)

S5 Fig Variant frequencies of HIV samples. (A) Shown are transition variant frequencies along the sequenced gag-pol region of HIV, with variant frequencies lower than 1% blurred, (B) Inferred haplotypes across all HIV samples. Details as in Figs 2 and 4, respectively.
(TIF)

S6 Fig Variant frequencies of RSV samples. (A) Shown are transition variant frequencies along the sequenced regions of RSV, with variant frequencies lower than 1% blurred. Dashed lines correspond to 1/# barcodes, theoretically the lower limit of detection. De facto the number of barcodes counted is a lower limit of the actual number of barcodes sequenced (see S1 Text), and we further note that the two amplicons of RSV (F/G gene, coordinates ~4640–7500, and L gene, coordinates ~8452–15025), underwent differential amplification. RSV6, RSV10, RSV24 were removed from the analysis due to <300 genomes sequenced. (B) Inferred haplotypes across all RSV samples. Notably RSV13 bears a haplotype with various different types of mutations, suggesting it may have an additional founder. Details as in Figs 2 and 4, respectively.
(TIF)

S7 Fig Variant frequencies of CMV samples. (A) Shown are transition variant frequencies along the sequenced regions of CMV, with variant frequencies lower than 1% blurred. (B) Inferred haplotypes across all CMV samples. Details as in Figs 2 and 4, respectively.
(TIF)

S8 Fig Technical replicates of AccuNGS sequencing. Technical replicates of HIV9 sample, all sequenced with AccuNGS. The two left panels were performed using Primer IDs (pIDs) whereas the right panel represents no primer ID. All three replicates show an excess of G>A mutations. While variants frequencies differed between samples, π diversity estimates were consistent (see S1D Fig), and multiple haplotypes bearing many G>A mutations were inferred in all three replicates. The highest coverage was obtained for the left panel (~320,000), followed by the middle panel (~270,000) and lastly by the right panel (~100,000). Higher coverage and a larger number of genomes sequenced on the left panel are likely responsible for lower frequency errors, in line with S2 Fig.
(TIF)
