# Supplementary material for: Drivers of within-host genetic diversity in acute infections of viruses
Source: PLoS Pathog. 2020 Nov 4;16(11):e1009029. doi: 10.1371/journal.ppat.1009029 (PMC7668575; doi:10.1371/journal.ppat.1009029)
Supplement: S1 Text — (DOCX) [file ppat.1009029.s001.docx]

**AccuNGS error landscape at the DNA level.** We began by measuring the error rate of the baseline AccuNGS protocol when tested on high volume homogenous DNA, and compared it to the results of the typical NGS protocol used when less emphasis is put on the fidelity of the process. Reassuringly, AccuNGS showed a significant improvement of one to two orders of magnitude over the standard sequencing protocol for most types of mutation (Fig. S1). We were able show that under these conditions of high volume DNA, errors are not likely derived from the library preparation steps including (i) PCR, (ii) gel extraction, (iii) DNA size selection (particularly UV light exposure), (iv) tagmentation during the NexteraXT DNA library preparation kit (Illumina), (v) alternative PCR cleanup procedures (magnetic beads extraction and using the Exosap cleanup reagent), (vi) the bacteria used to grow the plasmid, (vii) the particular sequence of the plasmid (Fig. S1, and data not shown). Notably, in some samples we observed the potential effects of oxidation and/or deamination, the former consistent with elevated G>T and C>A in a particular context (Fig. S1C), and the latter with elevated C>T at linked sites (Fig. S3). This has led us to conclude that these particular types of mutations are unreliable, especially when inferring haplotypes.

We test whether the sequencing machine itself was responsible for the observed errors. We applied a very stringent quality filtering of Q38 on one of our very high coverage samples denoted as AmpR. We expected that this filtering would improve the results by the difference between twice Q30 (Q60, error probability of 1x10^-6^) and twice Q38 (Q76, error probability of ~2x10^-8^). This difference translates to an improvement which is far below our observed error rate and hence we did not expect to see any improvement. Surprisingly, we observed a significant reduction in the rates of errors for A:T>G:C miscalls, and a modest yet significant reduction for C:G>T:A miscalls (Fig. S1B). We hence concluded that the assumption that the Q-scores of overlapping reads are independent (Zhang, et al. 2014; Edgar and Flyvbjerg 2015) is an incorrect assumption, and that the sequencer itself is likely the major source of errors in AccuNGS, when run on high-volume DNA.

**AccuNGS error landscape at the RNA level.** When sequencing RNA, the mean error rates are expected to be higher than DNA, with the difference stemming from RNA samples handling and the additional reverse transcription reaction. Indeed, sequencing high-volume RNA demonstrated an increase in the error rates of all mutations this increase was not dramatic (Fig. S1C, Table S2).

Previous work has demonstrated that low input of clinical samples may affect the prevalence of false-positives in the sequencing data (McCrone and Lauring 2016). We therefore sought to evaluate the differences in outcome when using AccuNGS sequencing to sequence synthetic constructs resembling clinical samples. We performed in vitro transcription of two HIV plasmids, pLAI.2 and pNL4-3 and designed an array of samples where we mixed RNA from both plasmids, while varying both minor strain (plasmid) levels (0.01, 0.001, 0.0005, 0.0002 and 0.0001 frequency of minor strain, denoted as dilution) and sample volume (10,000 (L), 100,000 (M) and 1,000,000 (H) copies within the sample), each in three independent biological replicates, targeting the same region as in the DNA experiments (HIV integrase). All samples were primed with degenerate barcodes (primer-IDs, see below) used later for counting the number of sequenced genomes. Following sequencing and base-calling we obtained mutational landscapes of these samples. Spiked-in variants (i.e., variants that separate the two plasmids) are our true positives whereas all other variants are our false positives (FP) (corresponding to orange and blue in Figs. S2-3, respectively). While it is clear that FPs exist in all sequenced samples, the variance in FP frequencies varied substantially, and variance decreased as the volume of the sample was higher. Importantly, we saw that two inferences remained stable: first, pi diversity of FPs remained stable (Fig. S3A). Second, we noted that we were able to accurately detect the presence of the minor haplotype. We noted some FP haplotypes that contained multiple G>T/C>A/C>T mutations, or very short haplotypes (based on 2-3 linked mutations) (Fig. S3B,C). Other than those, our haplotype reconstruction method consistently detected the spiked-in haplotype, contingent on high sample volume and high enough coverage (Table S4). We noted some over-estimation of haplotype frequencies; we cannot rule out however that the original dilutions may have been slightly inaccurate, since measurements of input were often on the border of detection of the measurement machines.

**Quantifying the amount of templates.** We began by testing whether it would be possible to quantify the number of templates sequenced, using uniquely barcoded primers during the reverse transcription process (Kivioja, et al. 2011, Jabara, et al. 2011). For the RSV samples, a barcode was added during the RT step, yet two independent amplicons were generated for the F-G region, and for the L region. We note that due to the tagmentation protocol we use, that may “cut” a barcode into two, the number of unique primer IDs identified is a lower bound on the number of actually-sequenced templates.

**Contamination across samples**. Following our haplotype reconstruction procedure, we initially noted the putative presence of an additional very low frequency haplotype in many of the HIV samples, and in some of the RSV samples. When examining these haplotypes, it turned out that they were often identical to a consensus sequence of another sample. This led us to suspect contamination may have occurred during (a) one of the stages of the library preparation, or (b) during sequencing, or (c) during de-multiplexing, when each read is assigned to a sample. We were able to mostly rule out the latter, by testing the hamming distance of the barcodes of the contaminated reads to the expected barcode of the sample. Notably, we took utmost care to avoid contamination during library preparation, including the fact that when samples were run together on a gel, we added an empty lane between each pair of samples, and used a different knife to cut out each band. We thus computationally flag contaminated reads and remove them from all subsequent analyses. The existence of low-level contamination, in spite all of the precautions taken, led us to hypothesize that indices impurity, rather than direct physical cross-sample contamination, is a possible source for the observed contamination. Illumina indices, used to distinguish between multiplexed samples, are typically comprised 8-bases each for the Nextera XT tagmentation process AccuNGS uses. A sample in paired-end sequencing is resolved to a given sample if the sequenced i5 and i7 indices match the preconfigured pair. Our initial protocol allowed for reusing one index while changing the other, such that each i5-i7 pair is unique but some indices are shared individually. To test if cross-sample contamination is reduced, we designed a sequencing experiment where we used 10-bases long i5 and i7 indices, ensuring each unique i5 and i7 is used only once in the sequencing experiment. We sequenced multiple samples belonging to different genetic backgrounds that were prepared separately, such that any contamination is observable. Reassuringly, we did not find any evidence for cross-sample contamination in this experiment (data not shown). We therefore recommend varying both i5 and i7 indices for AccuNGS sequencing experiments containing multiplexed samples.

## References

Edgar RC, Flyvbjerg H. 2015. Error filtering, pair assembly and error correction for next-generation sequencing reads. Bioinformatics 31:3476-3482.

Jabara CB, Jones CD, Roach J, Anderson JA, Swanstrom R. 2011. Accurate sampling and deep sequencing of the HIV-1 protease gene using a Primer ID. Proc Natl Acad Sci U S A 108:20166-20171.

Kivioja T, Vaharautio A, Karlsson K, Bonke M, Enge M, Linnarsson S, Taipale J. 2011. Counting absolute numbers of molecules using unique molecular identifiers. Nat Methods 9:72-74.

McCrone JT, Lauring AS. 2016. Measurements of Intrahost Viral Diversity Are Extremely Sensitive to Systematic Errors in Variant Calling. J Virol 90:6884-6895.

Moscona R, Ram D, Wax M, Bucris E, Levy I, Mendelson E, Mor O. 2017. Comparison between next‐generation and Sanger‐based sequencing for the detection of transmitted drug‐resistance mutations among recently infected HIV‐1 patients in Israel, 2000–2014. J INT AIDS SOC 20.

Zhang J, Kobert K, Flouri T, Stamatakis A. 2014. PEAR: a fast and accurate Illumina Paired-End reAd mergeR. Bioinformatics 30:614-620.

Zhao L, Illingworth CJR. 2019. Measurements of intrahost viral diversity require an unbiased diversity metric. Virus Evol 5:vey041.
