# Supplementary figures and images for: Drivers of within-host genetic diversity in acute infections of viruses
Source: PLoS Pathog. 2020 Nov 4;16(11):e1009029. doi: 10.1371/journal.ppat.1009029 (PMC7668575; doi:10.1371/journal.ppat.1009029)

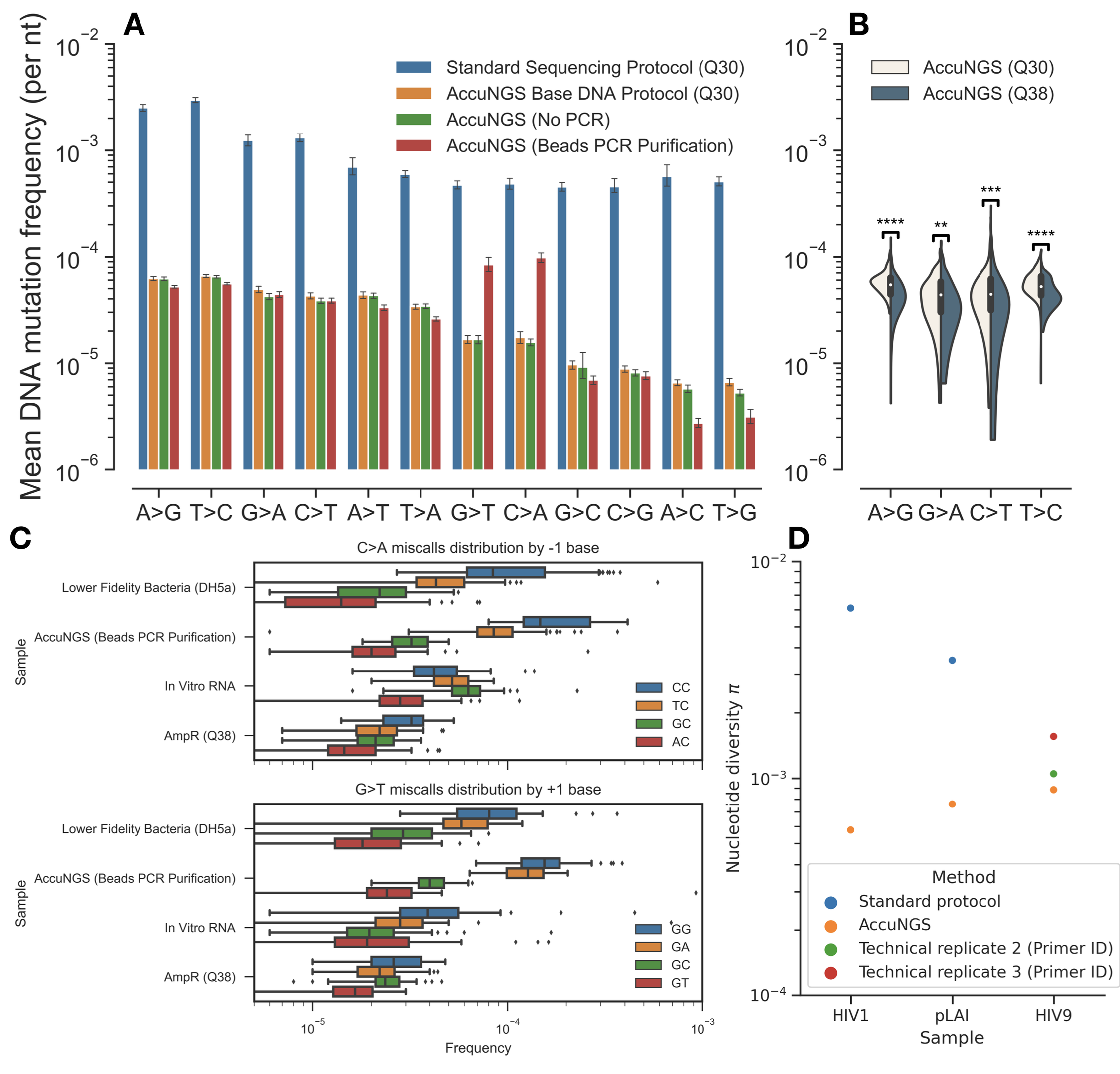

Supplement: S1 Fig — (A) AccuNGS dramatically reduces errors present in standard sequencing protocols by almost two orders of magnitude. For standard sequencing control, a standard homogeneous pLAI.2 control was taken from (Moscona, et al. 2017), and mutations were called without accounting for overlapping paired reads, while considering positions to analysis only if sequenced to at least 2,000x depth. PCR errors in AccuNGS are negligible (in average) based on the comparison of a PCR and PCR-free sample. Higher rates of G>T and C>A are likely indicative of oxidative stress. Error bars represent 95% confidence intervals around estimated mean values using 1,000 bootstrap repeats. (B) The effect of increasing the Q-score filtering threshold on AccuNGS error rates, presented for each type of transition error. A>G and T>C transitions show the most dramatic effect when increasing the Q-score filtering threshold. (C) Distributions of process errors potentially associated with oxidative damage (G:C>T:A). Notably in the In Vitro RNA sample, the C>A errors pattern is different from the pattern observed in other samples due to the single-stranded origin of this sample. Boxplots of errors per type of base changes are shown. Raw read bases were filtered when their average Q-score was less than Q30. (D) Comparison of pi diversity estimates using a standard sequencing approach versus AccuNGS. Substantial differences in pi diversity can be observed on the one biological sample (HIV1) that was sequenced with both methods, and on the pLAI plasmid sequenced with both methods. Also shown are technical replicates of sample HIV9 (see also S8 Fig). **p<0.01; ***p<0.001; ****p<0.0001. (TIF) [file ppat.1009029.s006.tif]

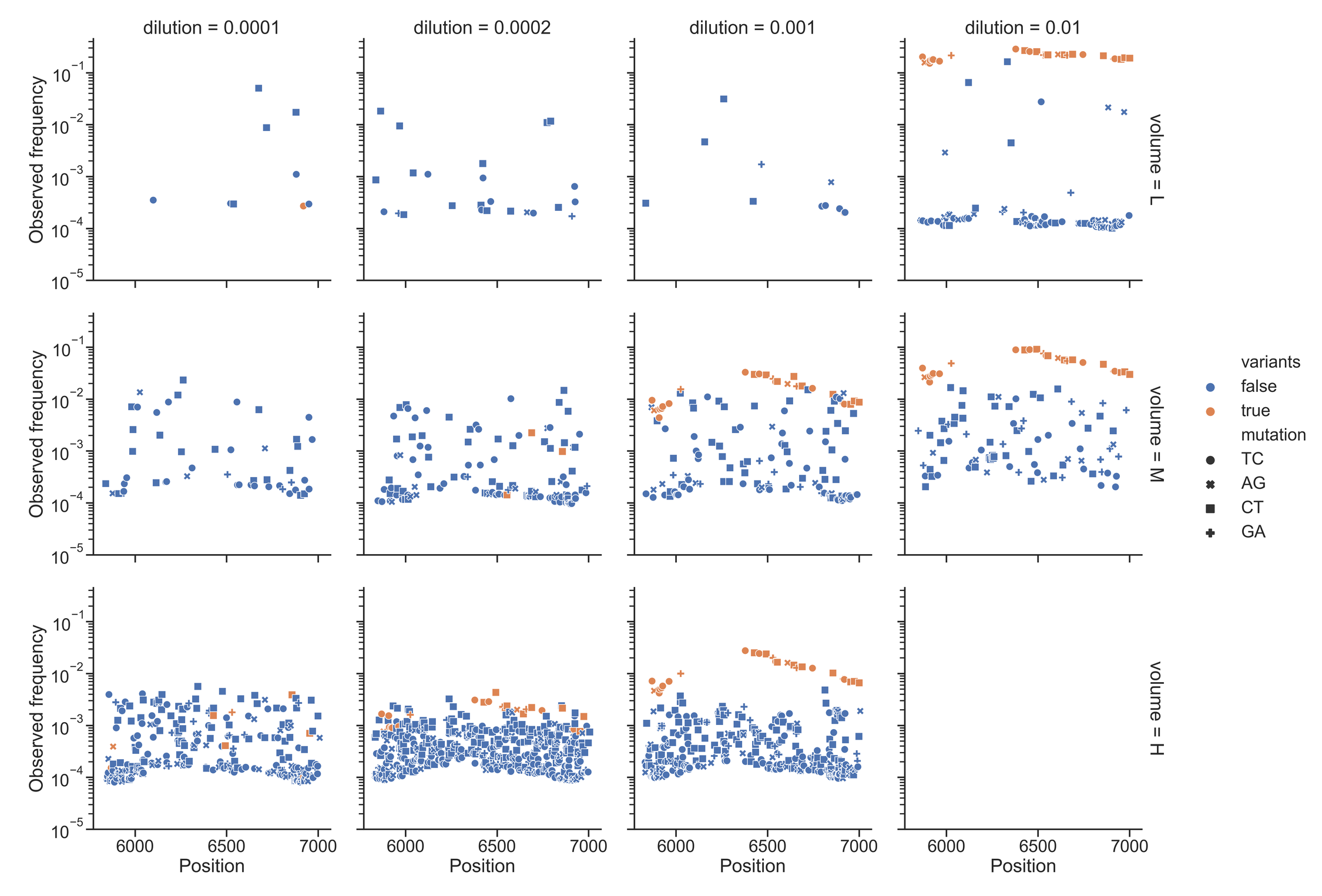

Supplement: S2 Fig — Three different volumes are presented, corresponding to the rows of the table: low viral load (total of 10,000 copies, label = L), medium viral load (total of 100,000 copies, label = M), and high viral load (total of 1M copies, label = H) (replicate B is shown). The expected frequency of the spiked-in minor haplotype/strain is shown at the top of each box. Blue variants were called from the dominant strain used (and hence false positives), and orange variants represent variants that distinguish the dominant and minor strain used (and hence true positives). The mean error rate based on the blue variants was around 5X10-4. However, the variance of errors was higher the lower the volume of the sample, leading to more high frequency errors in the lower volumes. The sequencing run for volume H and dilution 0.01 failed and hence is empty. This experiment was performed in three biological replicates (S4 Table); in this figure only replica B in shown. (TIF) [file ppat.1009029.s007.tif]

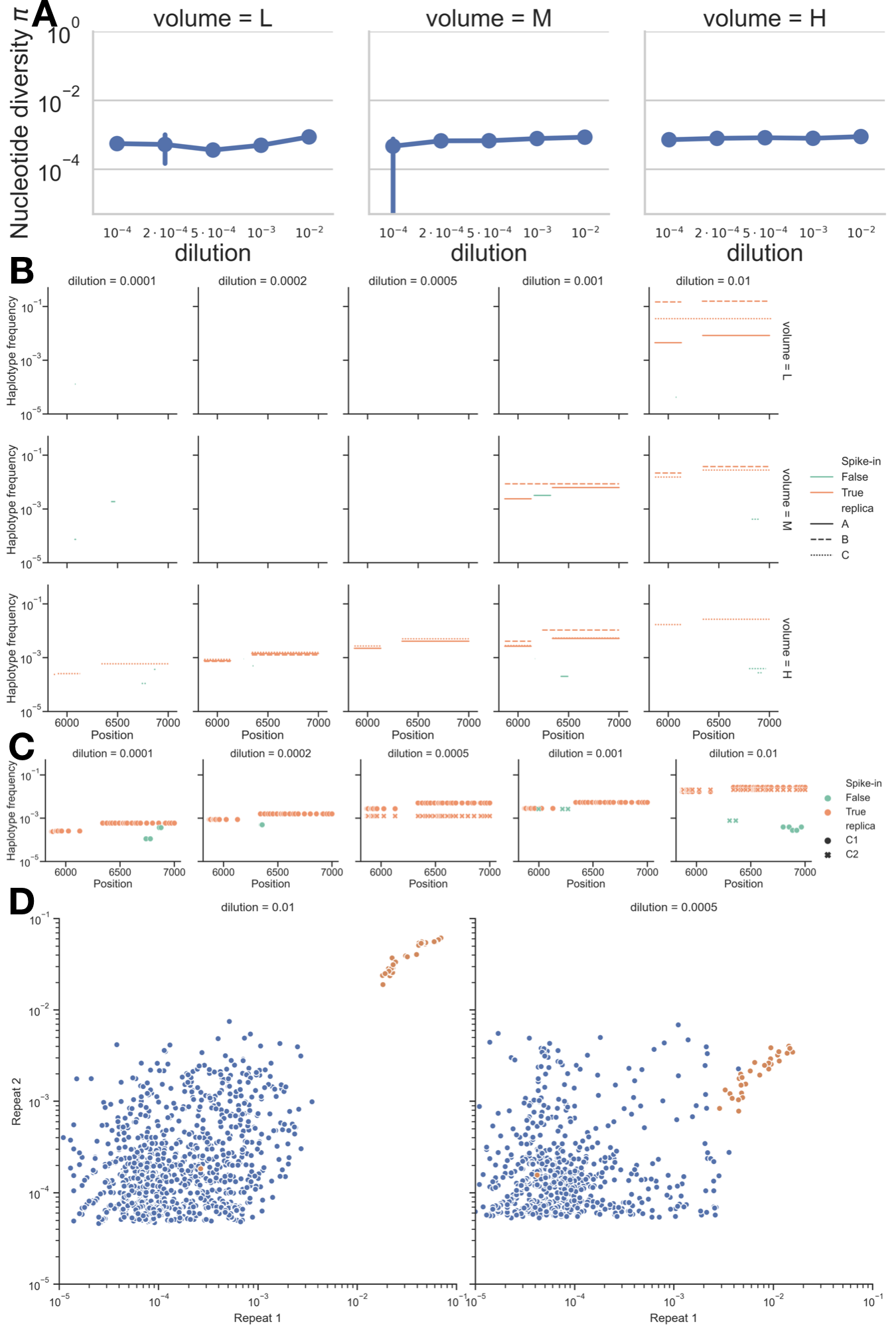

Supplement: S3 Fig — Details of the experiment and figure details as in S2 Fig. (A) Nucleotide diversity π measured on non-spiked-in variant positions shows a stable value around 5X10-4, that is independent of the number of input templates, and independent of the second spiked-in haplotype and its frequency. (B) Inferred minor haplotypes. Each type of line (solid, dashed, dotted) corresponds to a different independent biological replicate of the experiment. The higher the input volume, the more low- frequency haplotypes are captured. Haplotypes with multiple G>T/C>A/C>T variants removed. False positives were composed of at most three linked variants. (C) Inference of haplotypes on technical replicates (resequencing) of replicate C (circles, first technical replica; crosses, second replica). We noted X3 lower coverage in the second technical replicate, validated by less barcodes, leading to less inference of the spiked-in variant (Table S5). (D) Scatter-plot of mutation frequencies of the two technical replicates. Blue: false positives (errors), orange: true positives (true variants of spiked-in haplotypes). Reproducibility of true positives is demonstrated in C & D. (TIF) [file ppat.1009029.s008.tif]

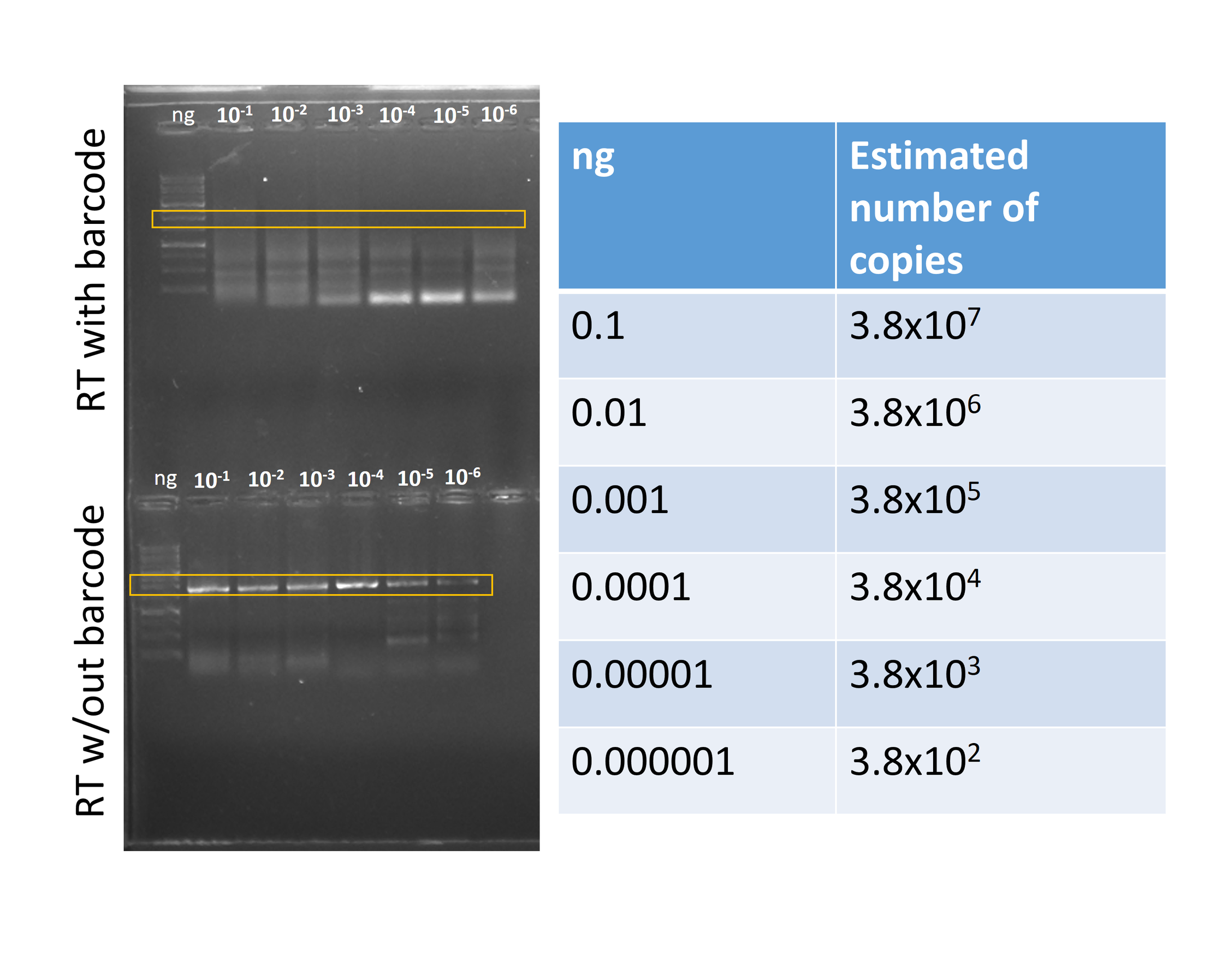

Supplement: S4 Fig — PCR results of serially diluted samples run with RT that includes a barcode (top) compared to RT without a barcode (bottom). The row corresponding to the estimated produced size (1935 bp with a barcode and 1860 bp without a barcode) is boxed. Estimated number of templates following dilution is shown on the right. (TIF) [file ppat.1009029.s009.tif]

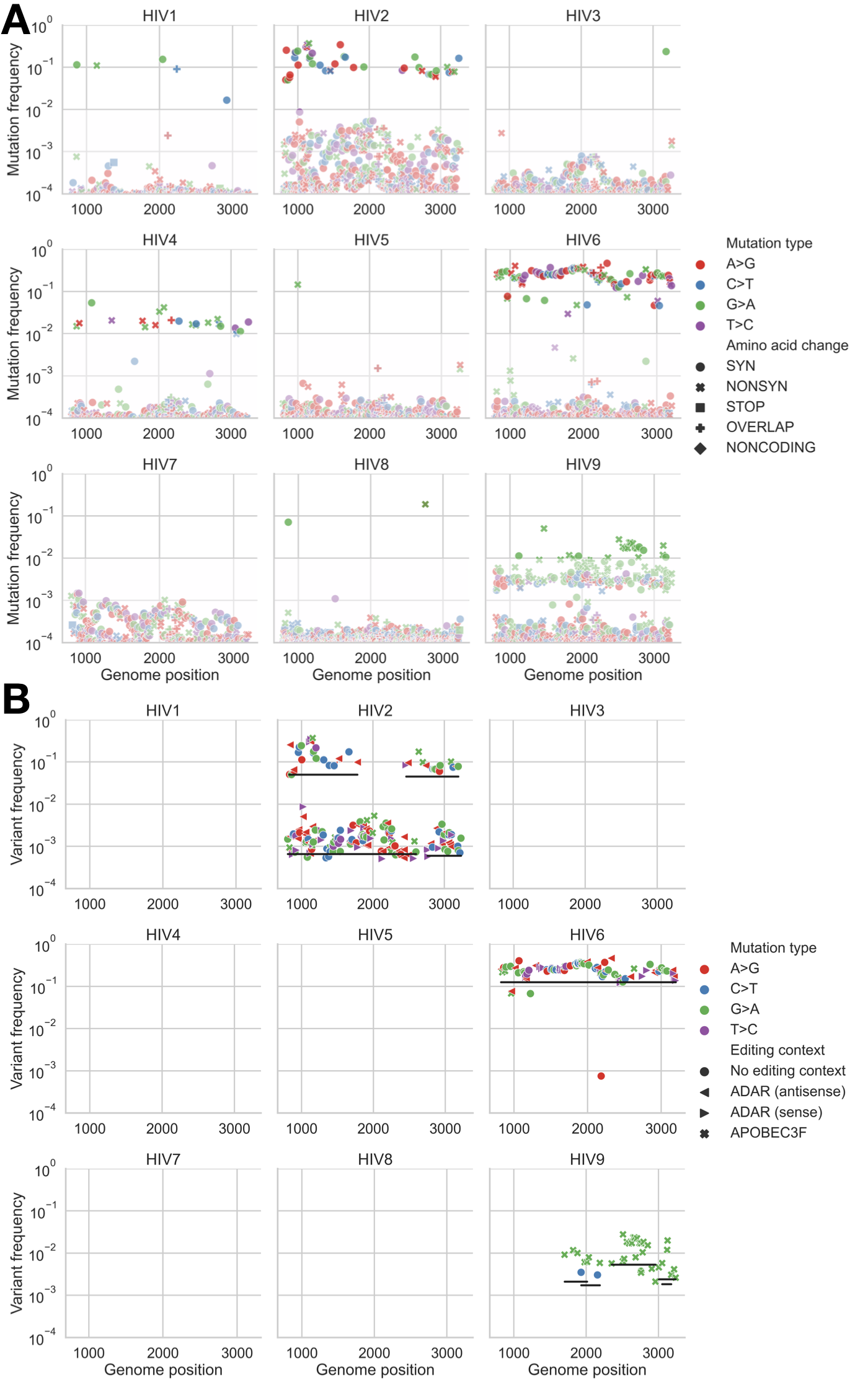

Supplement: S5 Fig — (A) Shown are transition variant frequencies along the sequenced gag-pol region of HIV, with variant frequencies lower than 1% blurred, (B) Inferred haplotypes across all HIV samples. Details as in Figs 2 and 4, respectively. (TIF) [file ppat.1009029.s010.tif]

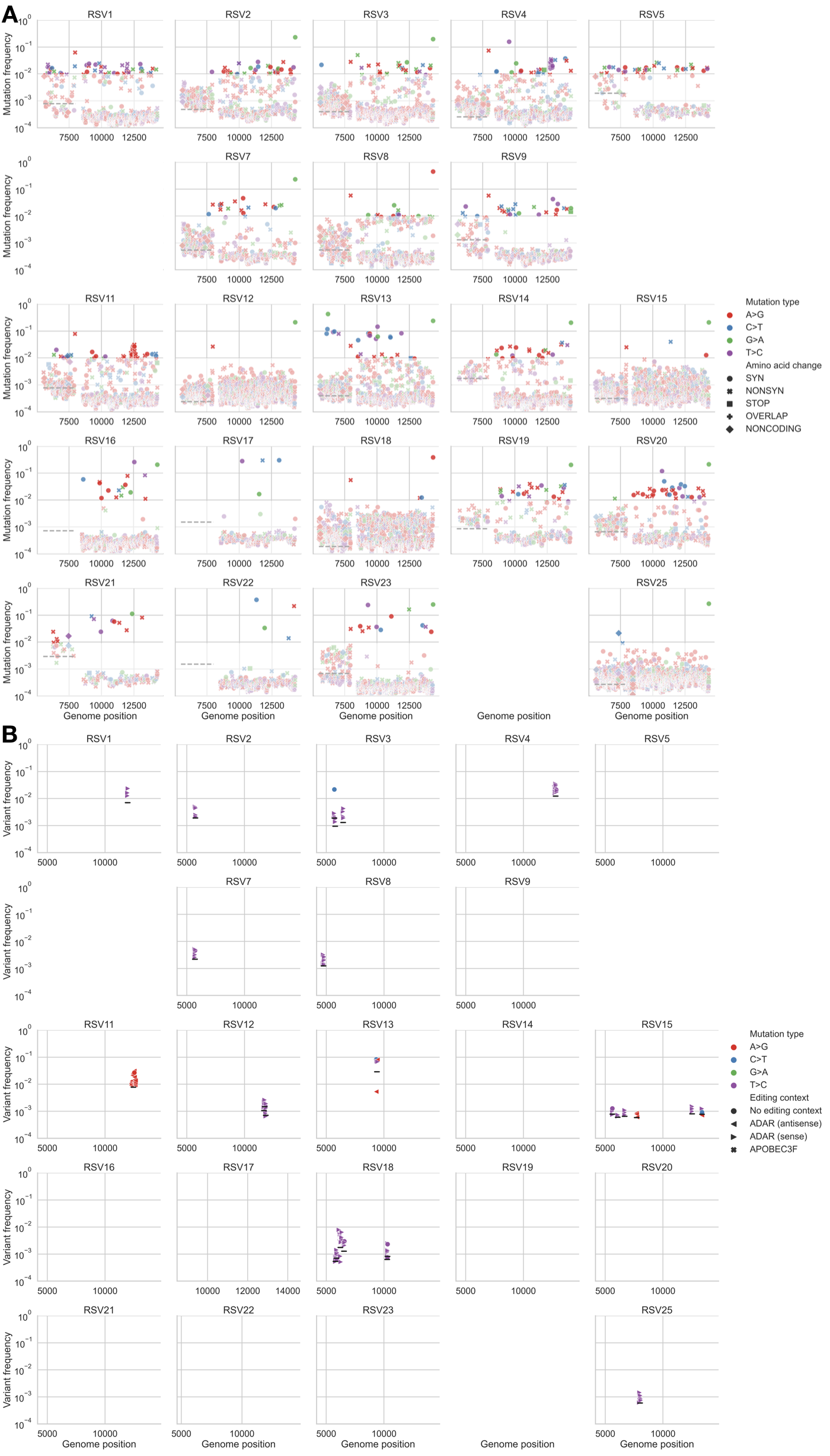

Supplement: S6 Fig — (A) Shown are transition variant frequencies along the sequenced regions of RSV, with variant frequencies lower than 1% blurred. Dashed lines correspond to 1/# barcodes, theoretically the lower limit of detection. De facto the number of barcodes counted is a lower limit of the actual number of barcodes sequenced (see S1 Text), and we further note that the two amplicons of RSV (F/G gene, coordinates ~4640–7500, and L gene, coordinates ~8452–15025), underwent differential amplification. RSV6, RSV10, RSV24 were removed from the analysis due to <300 genomes sequenced. (B) Inferred haplotypes across all RSV samples. Notably RSV13 bears a haplotype with various different types of mutations, suggesting it may have an additional founder. Details as in Figs 2 and 4, respectively. (TIF) [file ppat.1009029.s011.tif]

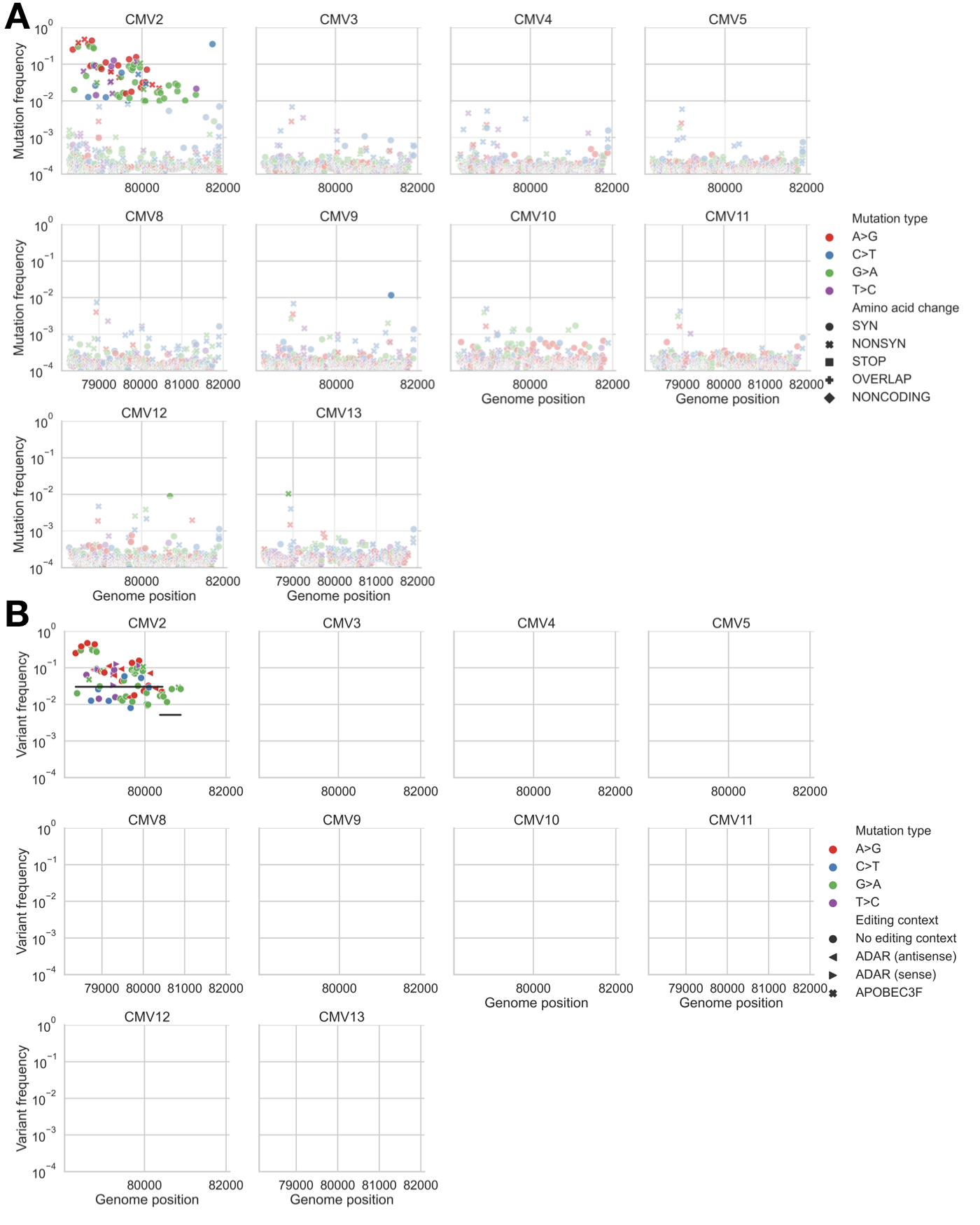

Supplement: S7 Fig — (A) Shown are transition variant frequencies along the sequenced regions of CMV, with variant frequencies lower than 1% blurred. (B) Inferred haplotypes across all CMV samples. Details as in Figs 2 and 4, respectively. (TIF) [file ppat.1009029.s012.tif]

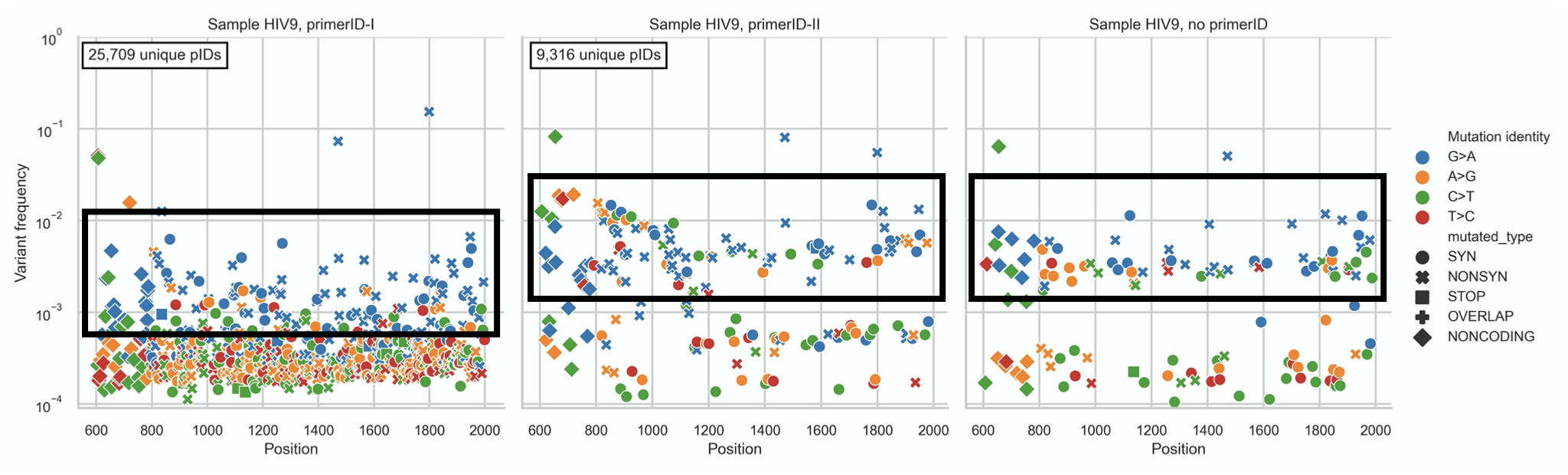

Supplement: S8 Fig — Technical replicates of HIV9 sample, all sequenced with AccuNGS. The two left panels were performed using Primer IDs (pIDs) whereas the right panel represents no primer ID. All three replicates show an excess of G>A mutations. While variants frequencies differed between samples, π diversity estimates were consistent (see S1D Fig), and multiple haplotypes bearing many G>A mutations were inferred in all three replicates. The highest coverage was obtained for the left panel (~320,000), followed by the middle panel (~270,000) and lastly by the right panel (~100,000). Higher coverage and a larger number of genomes sequenced on the left panel are likely responsible for lower frequency errors, in line with S2 Fig. (TIF) [file ppat.1009029.s013.tif]
