# Supplementary material for: Drivers of within-host genetic diversity in acute infections of viruses
Source: PLoS Pathog. 2020 Nov 4;16(11):e1009029. doi: 10.1371/journal.ppat.1009029 (PMC7668575; doi:10.1371/journal.ppat.1009029)
Supplement: Attachment — Submitted filename: reviewer response.docx [file ppat.1009029.s014.docx]

Dear editors,

This is a resubmission of manuscript PPATHOGENS-D-19-02056. We have profoundly revised in line with the constructive criticisms of the reviewers. We believe that this paper is now much improved and hope it meets the standards of the journal.

Sincerely,

Adi

Editorial review:

Thank you for the submission. There is editorial interest and interest among the reviewers in the topic and findings reported. However, reviewers 1 and 2 had serious concerns with the underlying data and validation of the method. Their concerns are overlapping and relate to replication and whether the plasmid controls adequately benchmark the detection thresholds (as opposed to RNA). Addressing these concerns seems beyond the scope of a "major revision."

We thank the editor and reviewers for important comments that have led us to significantly improve our understanding of the method. Most importantly, while we have now evaluated in depth the pros and cons of the sequencing method we developed, literally all of the original biological findings hold.

We summarize below the major changes we have made to the paper:

1. We have now benchmarked our method on in vitro transcribed RNA that mimics clinical samples of viruses (as opposed to high volume plasmid DNA/RNA). In line with previous reports, our results show that errors may be propagated in low viral loads. However, we show that our method is able to accurately detect the presence of extremely low frequency haplotypes, and to infer aggregated measures of diversity.
2. Accordingly, we have now revised the text to reflect these findings, and this is reflected in three paragraphs that were considerably modified, as well as supplementary figures that were added or removed. Importantly, **the vast majority** of the biological findings of the paper hold, since they are were based on aggregated measures of diversity and haplotype inference. We have removed one result on excess premature stop codons and deletions in RSV, yet this does not change any of the main conclusions of the paper.
3. The reviewers pointed out a potential discrepancy between mutation frequencies and number of templates sequenced. This was due to a mistake in presentation and interpretation, which we now clarify, and we thank them for pointing out this error.

Reviewer's Responses to Questions

Part I – Summary Please use this section to discuss strengths/weaknesses of study, novelty/significance, general execution and scholarship.

Reviewer #1: The manuscript of Gelbart et al describes a novel approach to viral genome sequencing allowing for an increased ability to detect low-frequency variation, applicable to patient samples.  The method is applied to sequence data, generating a few interesting results, for example, in characterising low within-host CMV diversity, but as presented the method is the key novelty here.  My concerns about the manuscript relate to the performance of the method.

Reviewer #2: The authors describe and validate a new viral deep-sequencing method called AccuNGS that is designed to accurately assess the frequencies of rare variants (as infrequent as 1:10,000 clinical viral populations. Their method uses high-fidelity and high-yield enzymes through the amplification process, and it also makes use of overlapping base pairs and base-specific error distributions to increase the accuracy with which variants can be identified. They apply AccuNGS to sequence clinical samples of HIV, RSV, and CMV. They find differences in the overall within-host genetic diversity of these viruses, and they also identify co-infection events, hypermutation, and defective viral genomes.

The manuscript describes interesting phenomena of co-infection and hypermutation that are worthy of attention. In particular, I think the inference of co-infecting viral haplotypes is nicely done. However, I am concerned about the validity of the sequencing methods described in the manuscript. In particular, I am worried that the authors have overlooked the effects of low viral load, which other studies (e.g. McCrone and Lauring, J. Virol. 2016; Grubaugh et al. Genome Biology, 2019) have demonstrated can significantly impact measurements of within-host genetic diversity. Several aspects of the presented data, including its low technical replicability, make me concerned that the authors have not adequately benchmarked their sequencing method for samples with low viral load, and I suspect that the AccuNGS method is not as accurate as the authors describe. These and other concerns are outlined in more detail below. Addressing these concerns would require significant additional benchmarking work. Alternatively, the authors could focus only on higher-frequency variants in their study (above 1-5%, as previous studies have focused on); for instance, their finding of a co-infecting HIV haplotype relied on variants at frequencies above 10%. Both of these routes might significantly alter the main conclusions of the manuscript.

Reviewer #3: See below

We thank the three reviewers for their detailed comments. We have performed extensive benchmarking of our method on synthetically created RNA that mimics key features of a population of viruses from clinical samples at various input levels (i.e., genome copy number). Our most important conclusion is that the vast majority of the biological findings in the paper hold, yet that the advantages of the method are different from what was originally described: (i) As the reviewers correctly pointed out, errors from RT or PCR may propagate when the input is low, and we have corrected the text to reflect this. (ii) However, the method is still able to accurately detect extremely low frequency haplotypes (1E-02 to 1E-04) , and to infer the level of genetic diversity in a sample. (iii) Our original inferences on differences in genetic diversity among samples, potential founder genotypes, and haplotypes associated with innate immune editing, all hold.

--------------------

Part II – Major Issues: Key Experiments Required for AcceptancePlease use this section to detail the key new experiments or modifications of existing experiments that should be absolutely required to validate study conclusions.Generally, there should be no more than 3 such required experiments or major modifications for a "Major Revision" recommendation. If more than 3 experiments are necessary to validate the study conclusions, then you are encouraged to recommend "Reject".

Reviewer #1:

1.1  I have some concerns about the ability of the method to characterise low-frequency variation.  For example, the technical replicate data from the HIV9 population shown in Figure S10 shows that results for the replicate datasets are quite different from one another.  Taking as an example a single variant, in site 1800 a variant is seen at around 12% on the left, around 7% in the centre, and at much lower frequency on the right.  Taking as another example lower frequency variation, the replicate samples have variously a small number, an intermediate number, and a high number of variants at around 0.3% minority allele frequency.  Where a single sample is split, and the number of viruses in the sample is high, the outcomes would be expected to be more similar, even with fairly standard sequencing methods.  Is there a reason for the large disagreements between replicates here?

We thank the reviewer for this comment, who noted correctly that individual low frequencies of mutations may be unreproducible. This comment together with other comments have led us to reevaluate our method as described above and in comment 2.1, and we find that variance is errors is negatively correlated with viral load (Fig. S2). We do see reproducibility in the detection of higher frequency mutations, yet our findings have led us to focus only on aggregated measures of diversity or on haplotypes, which are the only measures we report for the biological results section. We have changed the text to reflect the evaluation of the method:

“Recent work has suggested that for clinical samples with very limited input of viral genomes, accuracy may be hampered by sampling and by errors propagated across small numbers of genomes (McCrone and Lauring 2016; Illingworth, et al. 2017; Grubaugh, et al. 2019; Zhao and Illingworth 2019). We thus mimicked clinical samples of RNA viruses by synthetically creating samples with two different haplotypes at varying realistic viral loads, and tested the performance of AccuNGS on these samples. While we found that the *average* error rate of AccuNGS is low, our results showed that sampling, reverse transcription errors, and/or PCR biases may lead to a given variant being detected at a lower or higher frequency than it should be (Fig. S2). This was especially true when the number of input templates was low, and was likely due to early errors being propagated over a small number of templates. In fact, this leads us to caution against inferences made on individual mutation frequencies, especially those lower than ~1%, as noted previously (Grubaugh, et al. 2019) (Fig. S2). However, we could conclude that AccuNGS is useful for inferring aggregated measures of diversity, such as the total diversity in a sample or the average frequency of a group of mutations. Even more notable was the ability of AccuNGS to detect the presence of very low frequency haplotypes. Our results showed that AccuNGS can (a) detect π diversity with a limit of detection of 5x10^-4^, and (b) detect the presence of a haplotype as rare as 10^-4^, contingent on high enough coverage and template recovery (Fig. S3, supplementary text).“

1.2. I have a second concern about the claimed level of false-positive calls of variants, which seems to be around an order of magnitude higher than that identified for the plasmid data.  Specifically, Table S6 notes that 363 genomes were sequenced in HIV8, 115 for RSV6, and 65 for RSV10.  If I have understood this correctly, this implies that all variants detected at low frequencies in these samples are likely to be false positives, the cutoff representing the case in which a single virus carries a given variant.  However, the data presented in Figure S5 suggests that multiple variants at frequencies lower than the threshold were identified.  For example RSV10 has a large cloud of variation at around 10^{-3}, much lower than the nominal minimum frequency of 1/65.  This seems to imply that, where Figure S1A suggests an error rate between 10^{-4} and 10^{-6}, false positive identifications of varaints will often be observed at frequencies of around 10^{-3}.  Is this correct?

This result was due to erroneous presentation of the results. Our barcode was attached during RT only to the 5’ region of the RNA genome, and two amplicons were generated from the resulting DNA. The 5’ amplicon was amplified less efficiently than the second 3’ one. Focusing on the 5’ amplicon, we see a match between number of genomes and mutation frequencies. We also note that barcode counts are often underestimates of number of templates captured due to the tagmentation protocol we use. This is now explained in the supplementary text and in Fig. S6 (previously figure S5):

“…Dashed lines correspond to 1/# barcodes, theoretically the lower limit of detection. De facto the number of barcodes counted is a lower limit of the actual number of barcodes sequenced (see text above), and we further note that the two amplicons of RSV (F/G gene, F/G gene, coordinates ~4640-7500, and L gene, coordinates ~8452-15025), underwent differential amplification.”

1.3. While the distinction between characterising samples and characterising a population is acknowledged in the paragraph from line 124, there is some ambiguity on this point throughout the rest of the manuscript, with ‘sample’ and ‘population’ being used interchangeably, for example comparing lines 23-4 and 335-6.  I suggest referring to samples throughout, as the method deals with this step of the process.

We have replaced the word “population” with “sample” in all the relevant places.

Reviewer #2:

2.1. I am concerned that the authors have not adequately addressed the possible effects of low viral load on their sequencing results, even though other studies have demonstrated that low viral load can create many false-positive variants, likely through the accumulation of high-frequency variants during PCR. To address these concerns, the authors would need to estimate the error rates of their AccuNGS protocol using a range of starting template concentrations.

This is a valid concern that led us to reconsider the method and we thank the reviewer for raising this point. We have now extensively benchmarked the method on synthetic RNA mimicking clinical samples with varying starting template numbers (initial copy numbers): H (1M copies), M (100,000 copies), L (10,000 copies). These correspond to the range of initial copy numbers in our clinical samples (~11,000 to 1.1M). We further added a spiked-in additional haplotype (frequency between 1e-04 and 1e-02), and performed this in three independent biological replicates. One replicate underwent re-sequencing (technical replicate). As described above, this has led us to realize that RT and/or PCR errors may propagate with low copy number. This is now described in the main text and in two new supplementary figures S2 and S3. We have changed the text to reflect these shortcomings, but also emphasize the key strengths of our method: the ability to infer aggregated measures of diversity (e.g., pi diversity, which is insensitive to individual errors), and the ability to find extremely low frequency haplotypes.

Initial benchmarking of the AccuNGS method on plasmid controls indicated that error rates from library preparation were less than 1:10,000. However, these experiments appear to have been performed on plasmid controls (or in vitro transcribed RNA) that likely has very high copy number (I was unable to find the starting template concentration in the manuscript).

We have removed these data from the paper and replaced them with our benchmarking that is based on low input RNA, as described in the comment above.

In contrast, the authors sequence clinical viral samples with the number of sequenced templates ranging from 65 (as assessed by primer ID) to >10,000 (Table S6). (Note that the abstract reports that an average of >1000 viral templates was sequenced per sample, but this average does not well represent the range of the distribution.) For samples with fewer than 1000 sequenced templates, they still identify a large number of mutations in a 1:1000 to 1:10,000 frequency range. Some of these mutations might be genuine mutations present at low frequency due to inaccuracy in measuring exact frequencies, but in general,
variants with frequency below 1/(# of sequenced templates) are likely to be sequencing errors, regardless of the sequencing depth. I suspect that several other reported results that disagree with prior work (like the reported lack of effect of reverse transcription and PCR on error rates in Figure S1E and S1A) may also result from the fact that a large number of starting templates were probably used to benchmark the AccuNGS sequencing method. These concerns lead me to suspect that AccuNGS is not as accurate as the authors describe.

These concerns are addressed in the response to 1.2 above. We have corrected the abstract to reflect the range of templates we sequenced: “We applied AccuNGS to 43 clinical samples taken from early infections of the human-infecting viruses HIV, RSV and CMV, and sequenced each sample deeply, with hundreds to tens of thousands of virus templates sequenced per sample.”

2.2      The authors did not make much use of technical replicates to validate the accuracy of their variant-calling method, even though previous studies like McCrone and Lauring 2016 show that sequencing replicates are useful for eliminating false-positive variants. Their only data from technical replicates is concerning: in Figure S10, the left and middle panels showing technical replicates have very different apparent variant-frequency distributions, with almost all variants in the left panel falling between 1:1000 and 1:10,000 frequency and most variants in the middle panel falling between 1:100 and 1:1000 frequency. Variants above a frequency of 1:10 are also not observed consistently between the three replicates, making me even more concerned about whether many apparent, low-frequency variants are just errors from library preparation and sequencing. To address this concern, the authors should perform replicate sequencing of the clinical samples analyzed in this manuscript, and they should report what proportion of variants are observed in multiple sequencing replicates.

With respect to the HIV sample, we have now added inference that shows that the two key features of AccuNGS are reproducible in the three technical replicates of this sample: pi diversity, and the presence of a rare haplotype with abundant G>A mutations (Fig. S8) . We note that we could not perform resequencing of other clinical samples herein since we ran out of input material that was very low. We thus used our benchmarking data from synthetic RNA to verify reproducibility of our method. We found reproducibility of our haplotype inference, and also of frequencies higher than ~1%. This is now shown in figure S3C and S3D. We agree that individual mutation frequencies are often inaccurate and, consequently, now add a note of caution on inferences made from individual mutation frequencies: “In fact, this leads to caution against inferences made on individual mutation frequencies that are lower than ~1%, as noted previously (Grubaugh, et al. 2019) (Fig. S3).”.

Reviewer #3: This is an interesting and potentially important paper that describes a new approach genomics - AccuNGS - that uses a plasmid control (to a natural sample) to determine, with accuracy, levels of genetic variation in acute phase viruses. The authors find such variation to be extensive.

The authors have a generally done a good job with this paper and I have only relatively minor comments, although I don’t feel qualified to discuss some of the technical details of the sequencing methodology.

3.1 To my mind the weakest session is that on the transmission bottlenecks (in HIV). This is a highly contentious area and while I don’t disagree with what the authors have written, it is in reality little more than supposition. It needs more convincing data. I suggest this section is removed so as to avoid future criticism. Ultimately, they need to look at donor-recipient pairs and then to show how many minor variants are transmitted between them.

We agree and have toned down sections discussing this issue to reflect that this is only a hypothesis. For example, we now write in the discussion:

“Our results suggest that a prominent factor in determining the intra-host genetic diversity of a sample during acute infection is the number of diverse genotypes present in a sample, and in fact we find that the samples with the highest levels of diversity always show evidence for the existence of multiple genotypes. We suggest that these different genotypes reflect different founders/transmitted viruses, although definitive evidence for this would be from donor-recipient pairs.”

3.2 I don’t like this sentence: “they must develop their arsenal of genetic diversity to either escape from the immune system, evade drugs or adapt to a new environment (possibly even a new host (Parrish, et al. 2008)”. To me, this sounds group selectionist: that diversity is generated for future use. In fact, of course, most mutations are deleterious and inhibit fitness. This should be re-phrased.

We agree with the reviewer. This sentence (and in fact the entire section) has been removed.

3.3 Why a gamma distribution to describe miscall frequency?

Originally, this was because systematic miscalls have previously been shown to best fit a gamma distribution. However, due to changes in our understanding of the method we have now removed the fit to a gamma distribution, since it was based on high volume DNA, and since we have realized that individual mutation frequencies are unreliable (comments 1.1 and 2.1).

3.4 Table 1 is fascinating as it seems to imply that mutation frequencies are asymmetric: for example, T>C is twice C>T. If true, this is hugely important because it means that base frequencies are not stationary and that nucleotide substitution models (as used in phylogenetics) are erroneous. Am I interpreting this correctly?
This originally reflected errors in sequencing (composed of PCR errors and sequencing machine errors), rather than mutation rates of organisms, and thus these numbers are probably less relevant for substitution models.

3.5 Figure 2: is that a hyper-mutant I see in CMV?
We have inferred that this is an additional divergent genotype, and we now clarify this in the figure legend:
“Samples exemplify mixed genotype infections (HIV6, CMV2), mutation biases and presumable hypermutation via host editing (HIV9, RSV samples), and relatively homogenous populations (HIV3, CMV5, CMV4) (see text for details). “

3.6 The authors suggest that positive (immune) selection is in part responsible for the mutation frequencies seen in RSV. I’m struggling to see this as credible given the very short time-scale of infection. Could it really have changed allele frequencies? Is there another possible interpretation? How many replication cycles are we talking about? When does the immune system kick-in with RSV?
This is a good point, indeed these are short time-scales of days to several weeks (Table 1). We now acknowledge this limitation in the text:

“We did note that the immunogenic envelope proteins in this study (HIV Env and RSV G proteins), often known to be under positive selection (Seibert, et al. 1995; Nielsen and Yang 1998; Tan, et al. 2013), displayed on average higher diversity than the conserved genes (Fig. 2B). This suggests that despite the early stage of infection, some form of immune pressure may already be operating, yet this merits further investigation.”

3.7 The section on defective genomes is interesting. Could the authors use the number of stop codons to estimate the deleterious mutation rate? The idea is that, because they are lethal, stop codons must have arisen in the previous replication cycle.
We agree with the reviewer that this would be exceptionally useful. However, our overall error rate (~5E-4) is most likely higher than the deleterious mutation rate of these viruses (~1E-05).

--------------------

Part III – Minor Issues: Editorial and Data Presentation Modifications

Please use this section for editorial suggestions as well as relatively minor modifications of existing data that would enhance clarity.

Reviewer #1:

1. 4 Assuming that the above comments are addressed, can more be said about the ability of the method to identify single-locus variants?  My view is that the detection of variation at a level of 1:10,000 depends on a fairly special population structure, in which a large number of variants distinguish two haplotypes. The claim as written is OK, but more details would be valuable here.

This is an excellent question. We are indeed able to detect only haplotypes that are quite divergent from each other, which differ by at least two mutations for any window sized 250 bases (the length of a read). We write in the discussion:

“our data does not allow us to detect infection with multiple founders when they share very similar genotypes, since our haplotype reconstruction method relies on detecting short reads that share two or more mutations.”

1.5 In line 5 it is claimed that this is an in vivo sequencing method.  Under my interpretation of the phrase this is not literally the case, in so far as the sample is removed from the living organism before processing.

We have removed in vivo and now write: “Here, we present AccuNGS, a novel sequencing approach tailored for clinical samples that detects haplotypes as rare as 1:10,000”

1.6 Line 35: ‘key to the successful adaptation’

This sentence was removed.

1.7 Line 708: ‘Haplotype inference’ or ‘Inference of haplotypes’

Fixed

Reviewer #2: (No Response)

Reviewer #3: See above
