# Supplementary material for: Drivers of within-host genetic diversity in acute infections of viruses
Source: PLoS Pathog. 2020 Nov 4;16(11):e1009029. doi: 10.1371/journal.ppat.1009029 (PMC7668575; doi:10.1371/journal.ppat.1009029)
Supplement: Attachment — Submitted filename: reviewer response.docx [file ppat.1009029.s015.docx]

Editorial review:

Your manuscript was reviewed by two reviewers who raised major concerns previously. Both reported that the manuscript had been improved by the revisions and were positive about responsiveness to the prior reviews. However, both reviewers identified remaining concerns that need to be addressed. Most notably, both reviewers questioned the novelty of the sequencing method presented and indicated the need to revise presentation of the method. In addressing these concerns, we encourage you to strongly consider Reviewer 2’s suggestion to emphasize novel biological findings rather than placing the focus on the sequencing methodology.

We thank the editor and the reviewers for their in-depth comments. We have followed the advice of the editor and of Reviewer 2 (and 1), and placed the vast majority of the emphasis on the biological findings rather than the method.

Reviewer's Responses to Questions

**Part I - Summary**
Please use this section to discuss strengths/weaknesses of study, novelty/significance, general execution and scholarship.

Reviewer #1: This manuscript describes a novel approach to the sequencing of viral samples. Said approach is applied to a set of clinical viral samples, from which a number of biological conclusions are drawn. The manuscript contains some findings of interest, and is improved from the previous submission, though I retain a few concerns.

We thank the reviewer for the feedback. See below our responses to specific comments.

Reviewer #2: The revised manuscript presents a method to deep-sequence viruses from clinical samples and identify rare haplotypes. The authors apply the method to deep sequence clinical samples of HIV, RSV, and CMV. They compare overall levels of within-host genetic diversity, identify co-infection events, and find stretches of the genome that have undergone APOBEC3 hypermutation.

The manuscript includes substantial additional benchmarking work compared to the earlier version, and I appreciate the authors’ uncommon dedication to addressing comments from the previous round of review. To address my earlier concerns about how the sequencing method performs on samples of low viral load, the authors have performed benchmarking experiments by deep-sequencing various concentrations of in-vitro transcribed RNA. They find that individual variant frequencies are often unreliable, particularly at low viral loads, but they argue that aggregate diversity metrics like pi can still be reliably calculated (but see Major comment 3). Their biological findings remain consistent with the earlier manuscript version.

However, I have several remaining major concerns with the revised manuscript, mostly dealing with the presentation and interpretation of the sequencing approach. Most importantly, after carefully reviewing the authors’ new benchmarking data, I would suggest that the authors tone down their claims about the novelty of their sequencing method and focus instead on their biological results regarding co-infection of multiple haplotypes and hyperediting, as it is not clear to me that their sequencing method represents a substantial improvement over existing techniques. In my view, the manuscript’s interesting biological findings together with a lighter focus on the sequencing approach are already enough to merit publication.

We thank the reviewer for this positive feedback. As described below, we have now followed the reviewer’s suggestions.

**Part II – Major Issues: Key Experiments Required for Acceptance**
Please use this section to detail the key new experiments or modifications of existing experiments that should be absolutely required to validate study conclusions.

Generally, there should be no more than 3 such required experiments or major modifications for a "Major Revision" recommendation. If more than 3 experiments are necessary to validate the study conclusions, then you are encouraged to recommend "Reject".

Reviewer #1: 1. I have some concerns about the way in which the results of the manuscript are communicated. For example in the abstract it is claimed that this is a method designed for clinical samples that detects haplotypes as rare as 1:10,000. As I understand it, this is formally true, in the sense that the method can detect a second haplotype in the case that the sample contains two haplotypes which differ by multiple variants (I think at least 2 per 250 base pairs within some region of the genome). However, I disagree that this is representative of a typical clinical sample of an RNA virus as claimed on line 101. As noted for RSV, a clinical sample of a viral population can contain a very large number of underlying haplotypes, which may not greatly differ from one another. My understanding is that, when applied to such a sample, the method could potentially identify that the sample contains ‘two or more divergent haplotypes’ (line 243), but would not be able to characterise each of the multiple haplotypes down to a frequency of 1 in 10,000. The performance of the method is fairly described in the manuscript as a whole (e.g. lines 327-330). However, in that the 1:10000 thing only applies under highly specific circumstances, my view is that the abstract should include a more representative statement of the method’s performance.

We concur with the reviewer, and in line with the suggestions of reviewer 2 as well, we have completely removed from the abstract any references to the method, as well as to the haplotype reconstruction performance. In the introduction we now better describe this part of the work:

"a bioinformatics approach for the inference of divergent viral haplotypes from the sequencing data"

And in the results we write:

“…the method will only detect haplotypes that are quite divergent from each other, since it searches for two mutations per every 250 bases.”

2. As rightly noted in the introduction, a variety of attempts have been made to reduce the error rate of NGS sequencing. However, in the current manuscript I am left unclear what is the relative benefit brought by your method compared to the others. For example, Adam Lauring’s work on influenza sequencing describes populations with very low sequence diversity, suggesting an ability to characterise the diversity of viral populations down to a fairly low level. You report a limit of detection of \pi = 5 times 10^{-4}; is that as good, or better than previously published methods? Also, how does your output compare to not implementing the AccuNGS techniques? Of the results you describe in the manuscript, which could not have been obtained using a standard sequencing protocol?

To assess the difference between AccuNGS sequencing and standard sequencing, Fig.S1(D) now compares AccuNGS with standard sequencing that was performed in a clinical sequencing lab. The comparison includes the sequencing of the clinical sample denoted in our study as HIV1, and the p.LAI plasmid control sample. The inferred pi diversity was around an order of magnitude lower when sequenced using AccuNGS, reinforcing that sequencing errors are on average lower using our approach. The figure is shown below, and also shows that technical replicates do not show such large differences:

Additional advantages of our method are its high yield, which are what most likely allowed us to detect low frequency haplotypes with signatures of ADAR editing. We now write in the discussion: "Moreover, we suggest that the depth obtained by AccuNGS (number of templates sequenced) has allowed us to obtain many of the conclusions reached herein, especially the ability to detect low frequency haplotypes".

Finally, in line with the comments of reviewer 2, the focus of the paper is now almost exclusively on the biological results rather than the method.

3. In line 78 it is claimed that multiple transmitted founder viruses are a major contributor to genetic diversity in HIV. I think this refers to the time of acute infection, as later diversity is shaped by a variety of processes. In HIV it has been claimed that around 80% of infections are founded by a single viral particle (e.g. Joseph et al Nat Rev Microbiol 2015). Is it your belief that this is not correct? Given recent discussions in the literature your result on CMV is interesting; further clarity on HIV would be valuable here.

We have now clarified that our findings refer to early infection, and that our findings for HIV are in line previous findings showing that 80% of infections are due to single genotype infections:

“We demonstrate the role of multiple transmitted/founder viruses as major contributors to the genetic diversity in HIV and CMV during the early acute stage of an infection. “

And

“Accordingly, we found evidence for multiple founder infections in two of nine HIV-1 samples, in line with previous reports (Keele, et al. 2008).”

Reviewer #2: 1. I am concerned that the authors’ deep sequencing method is not sufficiently novel or improved to merit being named and being the main focus of the manuscript. I would suggest that the authors tone down their claims about the novelty of their sequencing method and refocus the manuscript around their biological results. Here are the reasons I think that this is the case:
a. The authors’ sequencing method differs from commonly used viral deep sequencing methodologies only in that it makes use of overlapping read pairs, which is not especially novel. Other procedures like using high-fidelity polymerases and minimizing loss of material between different steps of the protocol are already routine. On the analytical side, the main innovation is that the authors use read pair information to infer partial haplotypes, which other studies have also done (e.g. Illingworth Bioinformatics 2016, Xue et al. eLife 2017).
b. It is not clear to me that the authors’ method performs better than existing methods for viral deep sequencing in realistic scenarios. The authors present new benchmarking data for their deep sequencing method based on low-copy in-vitro transcribed RNA. Although they find in Figure S1 that their sequencing protocol has a low mutation frequency on high-copy DNA, the results in Figure S2 indicate that even under favorable circumstances (high template copy number), variants detected in the 10^-4 to 10^-2 frequency range are almost exclusively false-positive variants, in keeping with what most other viral deep sequencing studies have found (McCrone et al. J. Virol. 2016, Grubaugh et al. Genome Biol 2019).
c. It is not clear to me that the authors make use of the low-frequency variants and haplotypes that their sequencing method is supposed to be able to detect. The biological results in Figures 3 and 4 rely almost exclusively on variants at or above frequencies of about 0.5%, similar to most other viral deep sequencing studies. It’s not clear to me that Figure 2 would be substantially different if it used a variant frequency cutoff of 0.5% or 1%, like many other studies.
d. The authors write in the abstract and throughout the manuscript that AccuNGS can detect “haplotypes as rare as 1:10,000” (a revision from the previous version of the manuscript, where they wrote that AccuNGS could detect “variants as rare as 1:10,000”), but it is not clear to me that the authors’ data supports this conclusion. In the authors’ new benchmarking data on in-vitro transcribed RNA (Figure S2), when the true haplotype is present at a frequency of about 10^-4, the variants that make up the true haplotype appear to be distinguishable from the false-positive variants only if the true haplotype is known in advance; i.e. true-positive and false-positive variants overlap substantially in frequency when the true-positive haplotype is rare. Moreover, the true-positive haplotype is entirely undetectable at this frequency in samples with 10^4 (L) to 10^5 (M) RNA copies.

Altogether, I would suggest that the authors 1) remove “accurate” and “in vivo” (as mentioned by reviewer 1) from the title of the manuscript; 2) refrain from naming AccuNGS as a sequencing method distinct from other standard approaches; 3) significantly shorten the first section of the results, “An overview of the AccuNGS sequencing approach” and emphasize the substantial overlap of the authors’ sequencing approach with other, unnamed sequencing approaches; 4) remove all mentions of being able to detect “haplotypes as rare as 1:10,000,” which is not supported by the data.

We appreciate the raised concerns. We have performed the following changes:

- "accurate" and "in vivo" have now been removed from the title of the manuscript, and in fact the title has been shortened to “Drivers of within-host genetic diversity in acute infections of viruses“, reducing the focus from the method.
- The first section of the results outlining the sequencing approach is now much shorter and emphasizes previous novelties made towards accurate sequencing.
- We have completely removed the previous figure 1 describing the method. All in all, this section has been reduced from around 1.5 pages down to about half a page.
- All text discussing a haplotype detection threshold of 1:10,000 were removed as well.

We did choose to keep AccuNGS as a reference name for the combination of wet-lab techniques and especially the complementary codebase, as we believe this package of tools will be useful for future research, and this makes it easy to reference. We have further left the paragraph describing our results on errors propagated when copy number is low, as we felt these are important to re-iterate to the research community.

2. I would suggest that the authors revise their figures and analyses to include only variants above a threshold like 0.5%-1%. Figure S2 demonstrates that many false-positive variants are present in the frequency range from about 10^-4 to 10^-2 under realistic template concentrations. Given these results, I find it concerning to show figures like 3, S6, and S7 where variant frequencies are plotted in the 10^-4 to 10^-2 frequency range. The authors by and large do not interpret these variant frequencies anyway, and these variants most likely represent errors that have accumulated in library preparation.

Per the reviewer’s suggestion, figures 2 (previously 3), S5A, S6A, S7A now blur variant frequencies below 1%. Indeed, variants below 1% are more likely to be errors than variants above 1%, but we note that template recovery governs this phenomenon (Fig.S2). For many samples, there are many variants below 1% that we believe are very likely real, as we see the ADAR-mutations stretches in RSV15 in Fig.3 at ~0.1% frequency, and the A3G stretches in HIV9 at <1%.

3. I am concerned about the authors’ interpretation of nucleotide diversity in Figure 2. The authors cite Figure S3A, showing the sequencing of in-vitro transcribed RNA, and Figure S8, showing the sequencing of technical replicates, to justify their use of the pi statistic. However, in Figure S8, the pi statistic that they give for the technical replicates varies from 1.4e-4 to 9.6e-4, nearly an order of magnitude!
This range of estimates of pi that they give for technical replicates spans a large part of the range of genetic diversity estimates in Figure 2. I would suggest that the authors omit most of the paragraph starting with line 157, since it is already unclear how consistent the diversity differences are between these viruses. I also suggest that the authors revise lines like 122 that argue that diversity metrics are consistently calculated using their sequencing method.

We thank the reviewer for highlighting this inconsistency. This was a typing mistake, for which we apologize (consecutive lockdowns in Israel and lack of childcare). The data is now correctly presented in Fig.S1D (also shown below here, series of three dots on the right). The pi diversity values for the technical replicates are much more consistent than previously presented and we attribute observed differences to differences in number of genomes sequenced, and differences in coverage.

**Part III – Minor Issues: Editorial and Data Presentation Modifications**
Please use this section for editorial suggestions as well as relatively minor modifications of existing data that would enhance clarity.

Reviewer #1: (No Response)

Reviewer #2: 1. Lines 177f: The authors should acknowledge that the absence of the reverse transcription step is likely to contribute to the lower observed genetic diversity for CMV.

Text was added to clarify this. We now write:

“Of note, the presence of a reverse-transcription step during HIV and RSV sequencing may contribute to some of the observed differences between CMV and the RNA viruses.”

2. Line 367: typo

Fixed.

3. Figure S3: The viral load and # input genomes figures often have an unrealistic degree of precision.

The numbers of input genomes are now approximated, but we have left the viral load numbers unchanged, since these are external measurements reported by the hospitals.

4. The authors consistently use 40 cycles of amplification, but it would probably be prudent in future applications to use fewer to avoid template-switching that tends to occur after many cycles of PCR have taken place.

Indeed, template switching during PCR could reduce the linkage between two minor variants and lead to underestimation of haplotype frequencies. This is most prominent when performing nested PCR, but not necessarily with single PCR (Zanini et al. Virus Research 2017). In our experiments with two “strains” of synthetic DNA/RNA, we saw no evidence for template switching, despite 40 cycles of PCR. We have not added this to the text, in order to avoid placing additional focus on the method.
