# Supplementary material for: Drivers of within-host genetic diversity in acute infections of viruses
Source: PLoS Pathog. 2020 Nov 4;16(11):e1009029. doi: 10.1371/journal.ppat.1009029 (PMC7668575; doi:10.1371/journal.ppat.1009029)
Supplement: Peer review history

# Author comment: Author response to previous submission

**DOI:** 10.1371/journal.ppat.1009029.r001

8 Jul 2020

Attachment Submitted filename: reviewer response.docx Click here for additional data file.

# Peer review: Decision Letter 0

**DOI:** 10.1371/journal.ppat.1009029.r002

**Authors:** Anice C. Lowen, Adam S. Lauring

1 Aug 2020

Dear Dr. Stern,

Thank you very much for submitting your manuscript "Accurate in vivo population sequencing uncovers drivers of within-host genetic diversity in viruses" for consideration at PLOS Pathogens. As with all papers reviewed by the journal, your manuscript was reviewed by members of the editorial board and by independent reviewers. Specifically, your manuscript was reviewed by two reviewers who raised major concerns previously. In light of the reviews (below this email), we would like to invite the resubmission of a significantly-revised version that takes into account the reviewers' comments.

Both reviewers reported that the manuscript had been improved by the revisions and were positive about responsiveness to the prior reviews. However, both identified remaining concerns that need to be addressed. Most notably, both reviewers questioned the novelty of the sequencing method presented and indicated a need to revise presentation of the method. In addressing these concerns, we encourage you to strongly consider Reviewer 2’s suggestion to emphasize novel biological findings rather than placing the focus on the sequencing methodology.

We cannot make any decision about publication until we have seen the revised manuscript and your response to the reviewers' comments. Your revised manuscript is also likely to be sent to reviewers for further evaluation.

When you are ready to resubmit, please upload the following:

[1] A letter containing a detailed list of your responses to the review comments and a description of the changes you have made in the manuscript. Please note while forming your response, if your article is accepted, you may have the opportunity to make the peer review history publicly available. The record will include editor decision letters (with reviews) and your responses to reviewer comments. If eligible, we will contact you to opt in or out.

[2] Two versions of the revised manuscript: one with either highlights or tracked changes denoting where the text has been changed; the other a clean version (uploaded as the manuscript file).

Important additional instructions are given below your reviewer comments.

Please prepare and submit your revised manuscript within 60 days. If you anticipate any delay, please let us know the expected resubmission date by replying to this email. Please note that revised manuscripts received after the 60-day due date may require evaluation and peer review similar to newly submitted manuscripts.

Thank you again for your submission. We hope that our editorial process has been constructive so far, and we welcome your feedback at any time. Please don't hesitate to contact us if you have any questions or comments.

Sincerely,

Anice C. Lowen

Associate Editor

PLOS Pathogens

Adam Lauring

Section Editor

PLOS Pathogens

Kasturi Haldar

Editor-in-Chief

PLOS Pathogens

​orcid.org/0000-0001-5065-158X

Michael Malim

Editor-in-Chief

PLOS Pathogens

orcid.org/0000-0002-7699-2064

***********************

Your manuscript was reviewed by two reviewers who raised major concerns previously. Both reported that the manuscript had been improved by the revisions and were positive about responsiveness to the prior reviews. However, both reviewers identified remaining concerns that need to be addressed. Most notably, both reviewers questioned the novelty of the sequencing method presented and indicated the need to revise presentation of the method. In addressing these concerns, we encourage you to strongly consider Reviewer 2’s suggestion to emphasize novel biological findings rather than placing the focus on the sequencing methodology.

Reviewer's Responses to Questions

**Part I - Summary**

Please use this section to discuss strengths/weaknesses of study, novelty/significance, general execution and scholarship.

Reviewer #1: This manuscript describes a novel approach to the sequencing of viral samples. Said approach is applied to a set of clinical viral samples, from which a number of biological conclusions are drawn. The manuscript contains some findings of interest, and is improved from the previous submission, though I retain a few concerns.

Reviewer #2: The revised manuscript presents a method to deep-sequence viruses from clinical samples and identify rare haplotypes. The authors apply the method to deep sequence clinical samples of HIV, RSV, and CMV. They compare overall levels of within-host genetic diversity, identify co-infection events, and find stretches of the genome that have undergone APOBEC3 hypermutation.

The manuscript includes substantial additional benchmarking work compared to the earlier version, and I appreciate the authors’ uncommon dedication to addressing comments from the previous round of review. To address my earlier concerns about how the sequencing method performs on samples of low viral load, the authors have performed benchmarking experiments by deep-sequencing various concentrations of in-vitro transcribed RNA. They find that individual variant frequencies are often unreliable, particularly at low viral loads, but they argue that aggregate diversity metrics like pi can still be reliably calculated (but see Major comment 3). Their biological findings remain consistent with the earlier manuscript version.

However, I have several remaining major concerns with the revised manuscript, mostly dealing with the presentation and interpretation of the sequencing approach. Most importantly, after carefully reviewing the authors’ new benchmarking data, I would suggest that the authors tone down their claims about the novelty of their sequencing method and focus instead on their biological results regarding co-infection of multiple haplotypes and hyperediting, as it is not clear to me that their sequencing method represents a substantial improvement over existing techniques. In my view, the manuscript’s interesting biological findings together with a lighter focus on the sequencing approach are already enough to merit publication.

**********

**Part II – Major Issues: Key Experiments Required for Acceptance**

Please use this section to detail the key new experiments or modifications of existing experiments that should be absolutely required to validate study conclusions.

Generally, there should be no more than 3 such required experiments or major modifications for a "Major Revision" recommendation. If more than 3 experiments are necessary to validate the study conclusions, then you are encouraged to recommend "Reject".

Reviewer #1: 1. I have some concerns about the way in which the results of the manuscript are communicated. For example in the abstract it is claimed that this is a method designed for clinical samples that detects haplotypes as rare as 1:10,000. As I understand it, this is formally true, in the sense that the method can detect a second haplotype in the case that the sample contains two haplotypes which differ by multiple variants (I think at least 2 per 250 base pairs within some region of the genome). However, I disagree that this is representative of a typical clinical sample of an RNA virus as claimed on line 101. As noted for RSV, a clinical sample of a viral population can contain a very large number of underlying haplotypes, which may not greatly differ from one another. My understanding is that, when applied to such a sample, the method could potentially identify that the sample contains ‘two or more divergent haplotypes’ (line 243), but would not be able to characterise each of the multiple haplotypes down to a frequency of 1 in 10,000. The performance of the method is fairly described in the manuscript as a whole (e.g. lines 327-330). However, in that the 1:10000 thing only applies under highly specific circumstances, my view is that the abstract should include a more representative statement of the method’s performance.

2. As rightly noted in the introduction, a variety of attempts have been made to reduce the error rate of NGS sequencing. However, in the current manuscript I am left unclear what is the relative benefit brought by your method compared to the others. For example, Adam Lauring’s work on influenza sequencing describes populations with very low sequence diversity, suggesting an ability to characterise the diversity of viral populations down to a fairly low level. You report a limit of detection of \\pi = 5 times 10^{-4}; is that as good, or better than previously published methods? Also, how does your output compare to not implementing the AccuNGS techniques? Of the results you describe in the manuscript, which could not have been obtained using a standard sequencing protocol?

3. In line 78 it is claimed that multiple transmitted founder viruses are a major contributor to genetic diversity in HIV. I think this refers to the time of acute infection, as later diversity is shaped by a variety of processes. In HIV it has been claimed that around 80% of infections are founded by a single viral particle (e.g. Joseph et al Nat Rev Microbiol 2015). Is it your belief that this is not correct? Given recent discussions in the literature your result on CMV is interesting; further clarity on HIV would be valuable here.

Reviewer #2: 1. I am concerned that the authors’ deep sequencing method is not sufficiently novel or improved to merit being named and being the main focus of the manuscript. I would suggest that the authors tone down their claims about the novelty of their sequencing method and refocus the manuscript around their biological results. Here are the reasons I think that this is the case:

a. The authors’ sequencing method differs from commonly used viral deep sequencing methodologies only in that it makes use of overlapping read pairs, which is not especially novel. Other procedures like using high-fidelity polymerases and minimizing loss of material between different steps of the protocol are already routine. On the analytical side, the main innovation is that the authors use read pair information to infer partial haplotypes, which other studies have also done (e.g. Illingworth Bioinformatics 2016, Xue et al. eLife 2017).

b. It is not clear to me that the authors’ method performs better than existing methods for viral deep sequencing in realistic scenarios. The authors present new benchmarking data for their deep sequencing method based on low-copy in-vitro transcribed RNA. Although they find in Figure S1 that their sequencing protocol has a low mutation frequency on high-copy DNA, the results in Figure S2 indicate that even under favorable circumstances (high template copy number), variants detected in the 10^-4 to 10^-2 frequency range are almost exclusively false-positive variants, in keeping with what most other viral deep sequencing studies have found (McCrone et al. J. Virol. 2016, Grubaugh et al. Genome Biol 2019).

c. It is not clear to me that the authors make use of the low-frequency variants and haplotypes that their sequencing method is supposed to be able to detect. The biological results in Figures 3 and 4 rely almost exclusively on variants at or above frequencies of about 0.5%, similar to most other viral deep sequencing studies. It’s not clear to me that Figure 2 would be substantially different if it used a variant frequency cutoff of 0.5% or 1%, like many other studies.

d. The authors write in the abstract and throughout the manuscript that AccuNGS can detect “haplotypes as rare as 1:10,000” (a revision from the previous version of the manuscript, where they wrote that AccuNGS could detect “variants as rare as 1:10,000”), but it is not clear to me that the authors’ data supports this conclusion. In the authors’ new benchmarking data on in-vitro transcribed RNA (Figure S2), when the true haplotype is present at a frequency of about 10^-4, the variants that make up the true haplotype appear to be distinguishable from the false-positive variants only if the true haplotype is known in advance; i.e. true-positive and false-positive variants overlap substantially in frequency when the true-positive haplotype is rare. Moreover, the true-positive haplotype is entirely undetectable at this frequency in samples with 10^4 (L) to 10^5 (M) RNA copies.

Altogether, I would suggest that the authors 1) remove “accurate” and “in vivo” (as mentioned by reviewer 1) from the title of the manuscript; 2) refrain from naming AccuNGS as a sequencing method distinct from other standard approaches; 3) significantly shorten the first section of the results, “An overview of the AccuNGS sequencing approach” and emphasize the substantial overlap of the authors’ sequencing approach with other, unnamed sequencing approaches; 4) remove all mentions of being able to detect “haplotypes as rare as 1:10,000,” which is not supported by the data.

2. I would suggest that the authors revise their figures and analyses to include only variants above a threshold like 0.5%-1%. Figure S2 demonstrates that many false-positive variants are present in the frequency range from about 10^-4 to 10^-2 under realistic template concentrations. Given these results, I find it concerning to show figures like 3, S6, and S7 where variant frequencies are plotted in the 10^-4 to 10^-2 frequency range. The authors by and large do not interpret these variant frequencies anyway, and these variants most likely represent errors that have accumulated in library preparation.

3. I am concerned about the authors’ interpretation of nucleotide diversity in Figure 2. The authors cite Figure S3A, showing the sequencing of in-vitro transcribed RNA, and Figure S8, showing the sequencing of technical replicates, to justify their use of the pi statistic. However, in Figure S8, the pi statistic that they give for the technical replicates varies from 1.4e-4 to 9.6e-4, nearly an order of magnitude!

This range of estimates of pi that they give for technical replicates spans a large part of the range of genetic diversity estimates in Figure 2. I would suggest that the authors omit most of the paragraph starting with line 157, since it is already unclear how consistent the diversity differences are between these viruses. I also suggest that the authors revise lines like 122 that argue that diversity metrics are consistently calculated using their sequencing method.

**********

**Part III – Minor Issues: Editorial and Data Presentation Modifications**

Please use this section for editorial suggestions as well as relatively minor modifications of existing data that would enhance clarity.

Reviewer #1: (No Response)

Reviewer #2: 1. Lines 177f: The authors should acknowledge that the absence of the reverse transcription step is likely to contribute to the lower observed genetic diversity for CMV.

2. Line 367: typo

3. Figure S3: The viral load and # input genomes figures often have an unrealistic degree of precision.

4. The authors consistently use 40 cycles of amplification, but it would probably be prudent in future applications to use fewer to avoid template-switching that tends to occur after many cycles of PCR have taken place.

**********

PLOS authors have the option to publish the peer review history of their article (what does this mean?). If published, this will include your full peer review and any attached files.

If you choose “no”, your identity will remain anonymous but your review may still be made public.

**Do you want your identity to be public for this peer review?** For information about this choice, including consent withdrawal, please see our Privacy Policy.

Reviewer #1: **Yes: **Chris Illingworth

Reviewer #2: No

Figure Files:

While revising your submission, please upload your figure files to the Preflight Analysis and Conversion Engine (PACE) digital diagnostic tool, https://pacev2.apexcovantage.com. PACE helps ensure that figures meet PLOS requirements. To use PACE, you must first register as a user. Then, login and navigate to the UPLOAD tab, where you will find detailed instructions on how to use the tool. If you encounter any issues or have any questions when using PACE, please email us at figures@plos.org.

Data Requirements:

Please note that, as a condition of publication, PLOS' data policy requires that you make available all data used to draw the conclusions outlined in your manuscript. Data must be deposited in an appropriate repository, included within the body of the manuscript, or uploaded as supporting information. This includes all numerical values that were used to generate graphs, histograms etc.. For an example see here on PLOS Biology: http://www.plosbiology.org/article/info%3Adoi%2F10.1371%2Fjournal.pbio.1001908#s5.

Reproducibility:

To enhance the reproducibility of your results, PLOS recommends that you deposit laboratory protocols in protocols.io, where a protocol can be assigned its own identifier (DOI) such that it can be cited independently in the future. For instructions see http://journals.plos.org/plospathogens/s/submission-guidelines#loc-materials-and-methods

# Author comment: Author response to Decision Letter 0

**DOI:** 10.1371/journal.ppat.1009029.r003

1 Oct 2020

Attachment Submitted filename: reviewer response.docx Click here for additional data file.

# Editor report: Decision Letter 1

**DOI:** 10.1371/journal.ppat.1009029.r004

**Authors:** Anice C. Lowen, Adam S. Lauring

4 Oct 2020

Dear Dr. Stern,

We are pleased to inform you that your manuscript 'Drivers of within-host genetic diversity in acute infections of viruses' has been provisionally accepted for publication in PLOS Pathogens.

Before your manuscript can be formally accepted you will need to complete some formatting changes, which you will receive in a follow up email. A member of our team will be in touch with a set of requests.

Please note that your manuscript will not be scheduled for publication until you have made the required changes, so a swift response is appreciated.

IMPORTANT: The editorial review process is now complete. PLOS will only permit corrections to spelling, formatting or significant scientific errors from this point onwards. Requests for major changes, or any which affect the scientific understanding of your work, will cause delays to the publication date of your manuscript.

Should you, your institution's press office or the journal office choose to press release your paper, you will automatically be opted out of early publication. We ask that you notify us now if you or your institution is planning to press release the article. All press must be co-ordinated with PLOS.

Thank you again for supporting Open Access publishing; we are looking forward to publishing your work in PLOS Pathogens.

Best regards,

Anice C. Lowen

Associate Editor

PLOS Pathogens

Adam Lauring

Section Editor

PLOS Pathogens

Kasturi Haldar

Editor-in-Chief

PLOS Pathogens

​orcid.org/0000-0001-5065-158X

Michael Malim

Editor-in-Chief

PLOS Pathogens

orcid.org/0000-0002-7699-2064

***********************************************************

Reviewer Comments (if any, and for reference):

# Editor report: Acceptance letter

**DOI:** 10.1371/journal.ppat.1009029.r005

**Authors:** Anice C. Lowen, Adam S. Lauring

23 Oct 2020

Dear Dr. Stern,

We are delighted to inform you that your manuscript, "Drivers of within-host genetic diversity in acute infections of viruses," has been formally accepted for publication in PLOS Pathogens.

We have now passed your article onto the PLOS Production Department who will complete the rest of the pre-publication process. All authors will receive a confirmation email upon publication.

The corresponding author will soon be receiving a typeset proof for review, to ensure errors have not been introduced during production. Please review the PDF proof of your manuscript carefully, as this is the last chance to correct any scientific or type-setting errors. Please note that major changes, or those which affect the scientific understanding of the work, will likely cause delays to the publication date of your manuscript. Note: Proofs for Front Matter articles (Pearls, Reviews, Opinions, etc...) are generated on a different schedule and may not be made available as quickly.

Soon after your final files are uploaded, the early version of your manuscript, if you opted to have an early version of your article, will be published online. The date of the early version will be your article's publication date. The final article will be published to the same URL, and all versions of the paper will be accessible to readers.

Thank you again for supporting open-access publishing; we are looking forward to publishing your work in PLOS Pathogens. 

Best regards,

Kasturi Haldar

Editor-in-Chief

PLOS Pathogens

​orcid.org/0000-0001-5065-158X

Michael Malim

Editor-in-Chief

PLOS Pathogens

orcid.org/0000-0002-7699-2064
